# Clinical Applications of Anterior Segment Optical Coherence Tomography: An Updated Review

**DOI:** 10.3390/diagnostics14020122

**Published:** 2024-01-05

**Authors:** Yu Jeat Chong, Matthew Azzopardi, Gulmeena Hussain, Alberto Recchioni, Jaishree Gandhewar, Constantinos Loizou, Ioannis Giachos, Ankur Barua, Darren S. J. Ting

**Affiliations:** 1 Birmingham and Midland Eye Centre, Birmingham B18 7QH, UK; a.recchioni@bham.ac.uk (A.R.); ioannis.giachos@nhs.net (I.G.); ankur.barua@nhs.net (A.B.); 2 Modality Ophthalmology, Modality Partnership, Birmingham B19 1BP, UK; cloizou@nhs.net; 3 Department of Ophthalmology, Royal Free Hospital, London NW3 2QG, UK; matthew.azzopardi.14@um.edu.mt; 4 University Hospitals Birmingham NHS Foundation Trust, Birmingham B15 2GW, UK; gulmeenahussain@doctors.org.uk; 5 Academic Unit of Ophthalmology, Institute of Inflammation and Ageing, University of Birmingham, Birmingham B15 2TT, UK; 6 New Cross Hospital, Royal Wolverhampton NHS Trust, Wolverhampton WV10 0QP, UK; jaishree.gandhewar@nhs.net; 7 Academic Ophthalmology, School of Medicine, University of Nottingham, Nottingham NG7 2RD, UK

**Keywords:** anterior segment, cornea, epithelial mapping, keratitis, keratoconus, keratoplasty, optical coherence tomography (OCT)

## Abstract

Since its introduction, optical coherence tomography (OCT) has revolutionized the field of ophthalmology and has now become an indispensable, noninvasive tool in daily practice. Most ophthalmologists are familiar with its use in the assessment and monitoring of retinal and optic nerve diseases. However, it also has important applications in the assessment of anterior segment structures, including the cornea, conjunctiva, sclera, anterior chamber, and iris, and has the potential to transform the clinical examination of these structures. In this review, we aim to provide a comprehensive overview of the potential clinical utility of anterior segment OCT (AS-OCT) for a wide range of anterior segment pathologies, such as conjunctival neoplasia, pterygium, scleritis, keratoconus, corneal dystrophies, and infectious/noninfectious keratitis. In addition, the clinical applications of AS-OCT (including epithelial mapping) in preoperative planning and postoperative monitoring for corneal and refractive surgeries are discussed.

## 1. Introduction

Advances in imaging have revolutionized the field of ophthalmology, transforming our understanding, diagnosis, and treatment of ophthalmic pathologies. Among all imaging tools, optical coherence tomography (OCT) represents one of the most significant, ground-breaking advances in ophthalmic imaging. Since its introduction in the late 1980s, OCT has evolved to become an indispensable, noninvasive tool for in vivo imaging of the ocular structures [1]. Nowadays, it is considered to be a key instrument in typical clinical settings, ranging from tertiary hospitals to optometry clinics in the community [2]. Most ophthalmologists would be familiar with its use in the assessment and monitoring of retinal and optic nerve diseases [3]. However, it also has important applications in the assessment of the anterior segment structures including the cornea, anterior chamber, and iris. The clinical importance and applications of anterior segment OCT (AS-OCT) was first described in 1994 by Izatt et al. [4]. Since then, AS-OCT has been further developed, with its function extended to a wide range of practical clinical applications.

The aim of this review is to highlight the current clinical applications of commercially available AS-OCT technologies as an integral diagnostic tool for important ocular surface pathologies. As AS-OCT becomes ubiquitous, it has the potential to revolutionize the clinical examination of anterior ocular structures, much like how the introduction of the slit lamp revolutionized the ocular examination in its time. In addition, the clinical applications of AS-OCT to other anterior segment structures such as the sclera and conjunctiva are covered, alongside AS-OCT applications for corneal and refractive surgeries. AS-OCT has also been shown to be a valuable diagnostic tool for assessing the anterior chamber angle, and iris pathologies and morphologies, though this is beyond the scope of our review [5,6]. Furthermore, there has been an increasing clinical and research interest in the use of AS-OCT angiography (AS-OCTA) for various pathologies, for which detailed information can be found in recent reviews [7,8].

## 2. Technical Aspects of AS-OCT

AS-OCT is a safe imaging modality, with no reported ocular side effects to the best of our knowledge. It creates a composite image from a series of axial scans (A scans) [9]. Longer wavelengths (usually around 1310 nm) are typically used for AS-OCT in comparison to retinal imaging (typically between 800 and 1050 nm); however, this varies, as shown in Table 1 below [10,11]. The depth of penetration is influenced by the light scattering caused by opaque structures. As there is less scattering at longer wavelengths, the longer the wavelength, the deeper the penetration into deeper tissue structures [9]. The axial resolution of AS-OCT ranges between 2 and 25 µm [9,12], with ultra-high-resolution OCT (UHR AS-OCT) typically referring to a resolution of less than 5 µm [13]. However, this is usually limited to research settings. 

Other important characteristics of AS-OCT to consider are scanning speed, acquisition time, and processing and saving times. A faster scanning speed tends to have a shorter image acquisition time, which clinically can be an important factor for patient comfort, especially for patients who find it difficult to position on the machine [10,14]. However, the capturing of large amounts of data may lead to slower processing and saving times, which might impact the clinician’s workflow. Table 1 illustrates the different types of commercially available AS-OCT machines. 

**Table 1:** Comparison among some of the commercially available anterior segment OCT machines.

Characteristic	Time-Domain OCT	Fourier-Domain OCT		Ultra-High-Resolution OCT
		Spectral-Domain OCT	Swept-Source OCT	
Examples in clinical use	1. Visante OCT (Carl Zeiss Meditec, Jena, Germany) 2. Heidelberg slit lamp OCT (Heidelberg Engineering, Heidelberg, Germany)	1. Spectralis (Heidelberg Engineering, Heidelberg, Germany) 2. iVue80 (Optovue, Fremont, CA, USA) 3. Cirrus OCT (Carl Zeiss Meditec, Jena, Germany)	1. Casia SS-1000 OCT (Tomey, Nagoya, Japan) 2. Triton OCT (Topcon Corporation, Tokyo, Japan) 3. Anterion OCT (Heidelberg Engineering, Heidelberg, Germany) ***	1. SOCT Copernicus HR (Optopol Technologies SA, Zawiercie, Poland)
Optical source	Superluminescent diode [5]	Superluminescent diode [5]	Swept-source laser [5]	Superluminescent diode [5]
Wavelength	1 = 1310 2 = 1310 nm [5]	1 = 820 nm 2 = 840 nm 3 = 840 nm [5]	1 = 1310 nm 2 = 1310 nm [5] 3 = 1300 nm [15]	1 = 850 nm [16]
Scan width	1 = 16 mm 2 = 15 mm [17,18,19]	1 = 6 mm 2 = 13 mm 3 = 6 mm [5]	1 = 16 mm 2 = 16 mm [5,20] 3 = 9 mm [15]	1 = 10 mm [16]
Scan depth	1 = 6 mm 2 = 7 mm [17,18,19]	1 = 2 mm 2 = 2–2.3 mm (retina) 3 = 2 mm [5,21]	1 = 6 mm 2 = 6 mm [5] 3 = 11 mm [15]	N/A **
Axial resolution *	1 = 18 μm 2 = >25 μm [17,18,19]	1 = 7 μm 2 = 5 μm 3 = 5 μm [5,21]	1 = 10 μm 2 = 8 μm [5] 3 = <10 μm [15]	1 = 3 μm [16]
Transverse resolution *	1 = 60 μm 2 = 20–100 μm [17,18,19]	1 = 20 μm 2 = 15 μm 3 = 15 μm [5,21]	1 = 30 μm 2 = 30 μm [5] 3 = <45 μm [22]	1 = 12–18 μm [16]
A-scan rate (scanning speed in scans per second)	1 = 2000 scans/s 2 = 200 scans/s [5]	1 = 40,000 scans/s 2 = 80,000 scans/s 3 = 27,000 scans/s [5,21]	1 = 30,000 scans/s 2 = 100,000 scans/s [5] 3 = 16,640 scans/s [15]	1 = 52,000 scans/s [16]

* Some measurements are specific to the posterior segment used and might vary for anterior segment OCT; ** not documented in the literature; *** technical specifications shown are for the ‘Cornea Application’. This table was adapted from Azzopardi et al. [23].

## 3. Corneal Anatomy on AS-OCT Imaging

**Figure 1:**
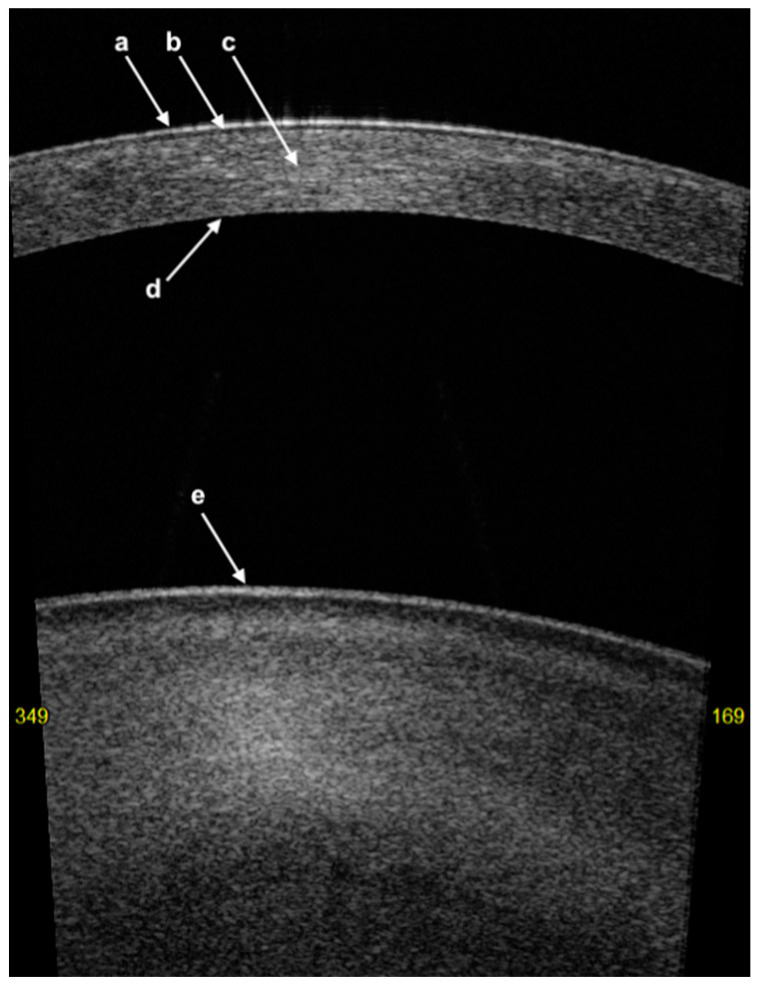
High-quality swept-source AS-OCT representation of a healthy cornea: (**a**) hyper-reflective tear film; (**b**) thin, hyporeflective corneal epithelium; (**c**) corneal stroma; (**d**) hyper-reflective pre-Descemet’s layer (or Dua’s layer)/Descemet membrane; (**e**) anterior surface of the crystalline lens.

On AS-OCT imaging, the layers of the cornea appear as follows (Figure 1):

1. The first hyper-reflective band represents the tear film with a thickness of less than 5 µm [24];
2. The first hyporeflective band represents the cornea epithelium with a thickness of between 50 and 70 µm;
3. Bowman’s layer (BL) is represented as a linear structure with similar hyper-reflectivity to that of the stroma;
4. The stroma has variable hyper-reflectivity with a thickness of around 500 µm;
5. The pre-Descemet’s layer (or Dua’s layer (PDL)) and Descemet membrane (DM) are shown as a hyper-reflective band of tissue. In the context of DM detachment (type 2 or mixed), PDL and DM may show as two separate hyper-reflective bands [25].

**Figure 2:**
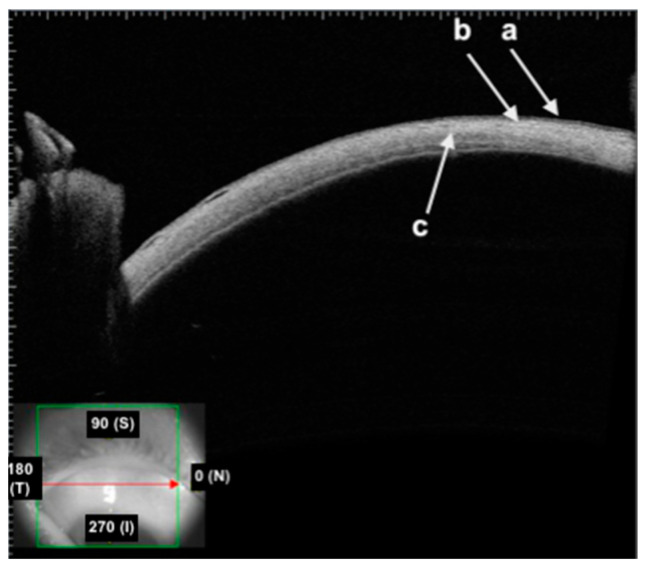
High-quality swept-source AS-OCT showing healthy conjunctiva: (**a**) hyporeflective conjunctival epithelium; (**b**) hyper-reflective subepithelial tissue of the substantia propria; (**c**) band of hyper-reflective scleral tissue.

On AS-OCT imaging, the layers of the bulbar conjunctiva appear as follows (Figure 2):

1. The first hyper-reflective and hyporeflective band both represent the conjunctival epithelium;
2. The second hyper-reflective band that underlies the conjunctival epithelium is the substantia propria;
3. The thick hyper-reflective band represents the sclera.

## 4. Clinical Applications for Conjunctival Diseases

### 4.1. Conjunctival Benign and Malignant Tumors

**Figure 3:**
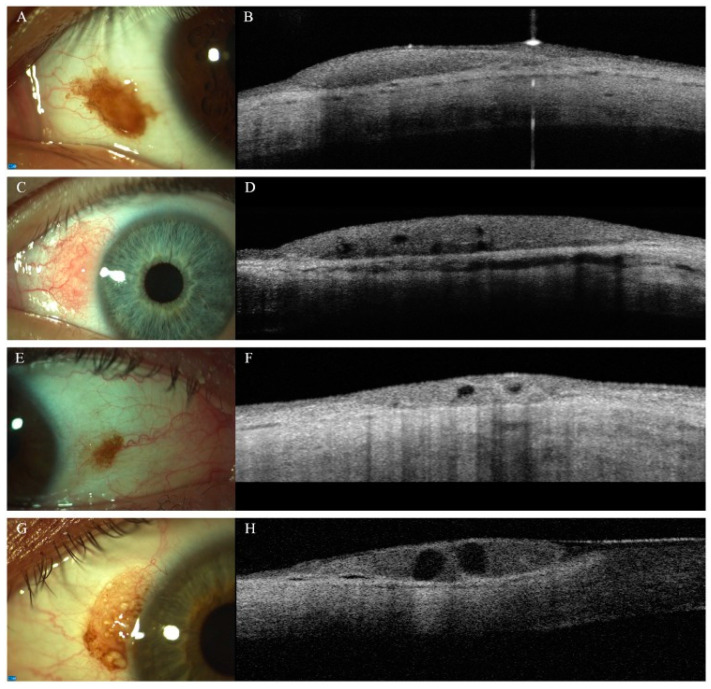
Clinico-morphological and AS-OCT features of conjunctival naevi: (**A**) pigmented lesion without cystic alterations on AS-OCT (**B**); (**C**) amelanotic naevus with macroscopically visible cysts, which are confirmed with AS-OCT examination (**D**); (**E**) small pigmented conjunctival naevus—cystic alterations are not be visible on slit-lamp examination, and intralesional cysts can be detected only with AS-OCT (**F**); (**G**) pigmented conjunctival naevus—intralesional cysts can be visualized with both slit-lamp examination and AS-OCT (**H**). (This figure was adapted from Vizvári et al. [29] and was reproduced unchanged under the terms of the Creative Commons (CC) BY 4.0 license https://creativecommons.org/licenses/by/4.0/, accessed on 28 September 2023).

Conjunctival melanocytic naevus is the most common tumor of the conjunctiva; its typical features include childhood onset, pigmentation, location within 1 mm of the cornea limbus in the nasal and temporal bulbar quadrant, and intralesional cysts <100 µm in diameter [26,27]. One of the important indicators of benign conjunctival naevus is the presence of intrinsic cysts (Figure 3), although small cysts can be difficult to detect even with other modalities such as ultrasound biomicroscopy, computer tomography, and magnetic resonance imaging (MRI) [28]. Studies have shown the superiority of AS-OCT in the provision of high-resolution imaging of conjunctival naevi, especially for the detection of intralesional cysts, with a higher sensitivity reported (61.4%) in comparison to that of slit-lamp (40.3%) and ultrasound biomicroscopy (28.5%) [29,30].

**Figure 4:**
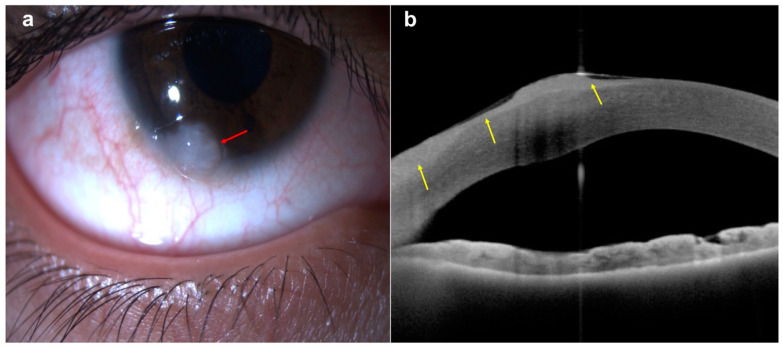
A case of conjunctival/corneal intraepithelial neoplasia: (**a**) slit-lamp photograph demonstrating a white, gelatinous lesion encroaching on the conjunctival, limbal, and peripheral corneal region (red arrow); (**b**) AS-OCT delineating the thickened epithelium at the conjunctival, limbal, and corneal regions (yellow arrows).

On the other hand, the top three primary malignancies of the ocular surface and conjunctiva are ocular surface squamous neoplasia (OSSN), melanoma, and lymphoma [31,32]. The typical clinical features of ocular surface squamous neoplasia (OSSN) are pearly grey mass with variable pigmentation, vascularity, and leukoplakia. On AS-OCT, the normal conjunctival epithelium can be abruptly disrupted by abnormal epithelium, characterized by thicker hyper-reflective lesions [33] (Figure 4).

In contrast to benign conjunctival naevi, the typical presentation of conjunctival melanoma is a thickened, elevated, pigmented lesion with feeder vessels, together with a surrounding area of melanosis [34,35]. It usually presents in adulthood instead of childhood. On AS-OCT, there is usually normal basal epithelium, with abnormal subepithelial hyper-reflectivity representing atypical melanocytes [36]. In these scenarios, AS-OCT technology is however limited by the challenge of penetrating thick pigmented lesions, given that the abnormality is subepithelial [35]. Penetration can be improved through the use of UHR AS-OCT, but this is not usually available outside research facilities [36].

**Figure 5:**
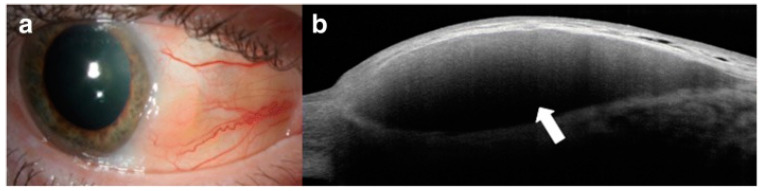
Slit-lamp photograph and AS-OCT of conjunctival lymphoma: (**a**) slit-lamp photograph of conjunctival lymphoma; (**b**) on AS-OCT, there is a homogeneous, dark, hyporeflective subepithelial lesion with smooth borders and overlying thin epithelium (arrow). The lesion contains monomorphic, stippled, dot-like infiltrates corresponding to the infiltration of monoclonal lymphocytes (this figure was adapted from Venkateswaran et al. [39] and was reproduced unchanged under the terms of the CC BY 4.0 license, https://creativecommons.org/licenses/by/4.0/, accessed on 28 September 2023).

Lastly, conjunctival lymphoma is characterized by a conjunctival mass, salmon-pink in color, smooth and multilobulated, with the presence of feeder vessels. It can also sometimes mimic follicular and papillary conjunctivitis [37]. On AS-OCT, it shows up as a homogenous, dark subepithelial lesion with smooth borders, containing monomorphic dot-like infiltrates [38] (Figure 5).

### 4.2. Pterygium, Pinguecula, Pseudopterygium, and Salzmann Nodular Degeneration

Pterygium is one of the commonest ocular surface diseases, resulting from the growth of subepithelial fibrovascular tissue originating from the bulbar conjunctiva, which eventually encroaches on the cornea, and is often associated with UV exposure [40,41]. Its prevalence is estimated to be up to 10 percent globally, with significant geographical variations [42]. Pinguecula, on the other hand, presents as a raised yellowish growth of the conjunctiva, with deposition of elastic-like material [43]. As it encroaches onto the cornea, it can transform into pterygium. The prevalence of pinguecula can be close to 50 percent in some populations [44,45,46,47]. Salzmann nodular degeneration (SND) is another slowly progressive, degenerative ocular surface disease characterized by whitish nodule(s) of the corneal subepithelial layer (anterior to the Bowman’s layer).

**Figure 6:**
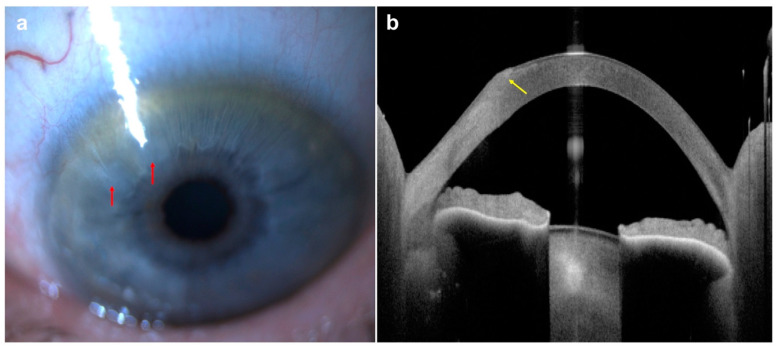
Salzmann nodular degeneration: (**a**) slit-lamp photograph demonstrating two Salzmann nodules on the cornea (red arrows); (**b**) AS-OCT demonstrating a corresponding hyper-reflective subepithelial lesion of the cornea (yellow arrow).

On AS-OCT, pterygium tissues appear as a subepithelial hyper-reflective lesion from the conjunctiva with extension into the cornea, with separation of the cornea epithelium from BM [48]. Pinguecula would have a similar appearance, only stopping short at the corneal limbus without extension into the cornea. Similarly, SND appears as a hyper-reflective lesion on the cornea limited to the subepithelial corneal layer (Figure 6).

In contrast with pterygium, pseudopterygium is characterized as a nonprogressive conjunctival adhesion to the peripheral cornea secondary to cornea limbal damage from chemical injury or chronic inflammation [49]. It can occur at any conjunctival quadrant, unlike pterygium, which has a predilection to arise from the nasal conjunctiva. Pseudopterygium is usually nonadherent to the limbus and has edges that can be elevated, under which a probe can be passed through. In clinical practice, this test might be difficult to perform due to patient discomfort. On AS-OCT, pseudopterygium is visualized as an overgrowing membrane that is not attached to the underlying cornea [48].

## 5. Clinical Applications for Scleral Diseases

### Episcleritis and Scleritis

Scleritis is a potentially sight-threatening inflammation of the sclera [50]. In anterior scleritis, there is involvement of both the superficial and deep episcleral capillary network. Comparatively, episcleritis is typically a self-limiting condition affecting only the superficial capillary network. While pain is the hallmark symptom of scleritis, clinical examination to differentiate between the two can be difficult due to overlapping features [51]. A common test used is topical 10% phenylephrine, which causes blanching of the superficial vessels in episcleritis. Other clinical differentiating signs include a distinct red hue in episcleritis versus a bluish-violet hue in scleritis [51]. Clinical assessment is however still subjective and not always straightforward.

**Figure 7:**
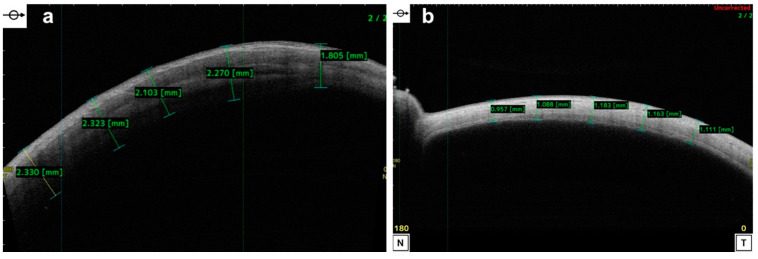
AS-OCT images of scleritis, comparing superior quadrant scleral thickness: (**a**) pretreatment; (**b**) post-treatment.

On AS-OCT, episcleritis tends to appear with increased episcleral and episcleral network thickness, with minimal changes in scleral thickness. However, the total combined thickness of the conjunctiva, episcleral, and sclera can be increased in both episcleritis and scleritis (Figure 7) [52]. The key difference that points toward a diagnosis of scleritis is an increase in hyporeflective spaces at variable depths, corresponding to dilated superficial and deep episcleral blood vessels [53]. Correlation with clinical exam is important, as greater dilated blood vessels on clinical exam reflect larger hyporeflective spaces on AS-OCT.

## 6. Clinical Applications for Corneal Diseases

### 6.1. Keratoconus

In 1844, the physician James Pickford wrote, ‘There is probably no disease to which the eye is subject, hitherto so rebellious to medicine, so intractable in its natures, and, at the same time, so fatal to vision, as conical cornea…the pathology and treatment of which are so little understood [54]’.

More than a century and a half later, we understand keratoconus as a noninflammatory bilateral, asymmetric ocular disease that causes astigmatism and impaired vision as a result of progressive corneal thinning and steepening [22,55]. Due to substantial global variation, the prevalence of keratoconus is estimated to be between 0.2 and 4790 per 100,000 people, with a predilection for people between their 20s and 30s [55]. Variability between different epidemiological studies have been attributed to differences in geography, ethnicity, keratoconus definition and diagnostic criteria, study design, and the age and cohort of subjects assessed [55].

The clinical features of keratoconus on slit-lamp examination have been well chronicled, including numerous clinical/eponymous signs such as a scissoring reflex during retinoscopy, Munson’s sign (deformation of the lower eyelid on downgaze due to protrusion of the cornea, Rizzuti’s sign (nasal limbus reflection that appears following the direction light at the temporal limbus), Fleischer’s ring (subepithelial deposition of iron oxide hemosiderin around the base of the cone), Vogt’s striae (vertical stress lines within the posterior stroma of the cornea that disappears with digital pressure), and oil droplet reflex (a dark shadow appearance in the midperiphery of the cornea on retroillumination) [56].

The early detection of keratoconus can lead to improved patient outcomes, with timely reviews to monitor disease progression and appropriate interventions with corneal cross-linking when indicated [57,58,59,60]. With certain machines, AS-OCT technology can generate a quantitative pachymetry map with parameters including keratometry, inferior–superior (I-S) dioptric value, and multivariate indexes such as KISA% [61]. In keratoconus, the typical characteristics include thinner-than-average corneas with inferior focal thinning [61]. The clinical use of AS-OCT-based pachymetry mapping is supported by the good intertest concordance with other modalities, including ultrasound pachymetry and Scheimpflug-based instruments such as the Pentacam (Oculus, Wetzlar, Germany) [62,63]. However, AS-OCT has been reported to have a better repeatability of the anterior corneal parameters in mild-to-moderate keratoconus as well as posterior corneal parameters in both normal and keratoconic eyes [64]. In addition, AS-OCT-based epithelial mapping has been increasingly shown to be a useful diagnostic test for detecting early keratoconus, as discussed later [63,65].

**Figure 8:**
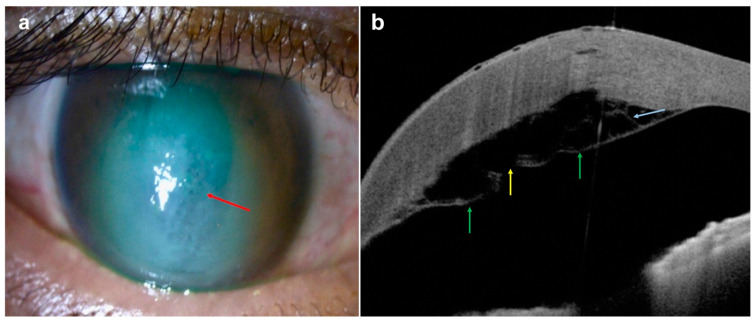
Acute corneal hydrops in advanced keratoconus: (**a**) slit-lamp photograph demonstrating a right acute corneal hydrops, characterized by significant central corneal edema, opacity, and bullae (red arrow); (**b**) AS-OCT demonstrating significant corneal edema with a break in pre-Descemet layer/Descemet membrane (PDL/DM; yellow arrow) and large type 1 PDL/DM detachment (green arrows). Attachment of posterior stromal fibers to PDL is visible (blue arrow).

One of the severe complications of keratoconus is the development of acute corneal hydrops, which is due to a spontaneous break in the PDL/DM, resulting in the imbibition of the anterior chamber aqueous fluid into the cornea stroma [66,67,68,69] (Figure 8). In serious cases of acute corneal hydrops, scar tissue can impair visual acuity and might necessitate treatment with scleral contact lenses or even corneal transplant [70,71]. On AS-OCT, the integrity of the PDL/DM can be visualized and characterized based on parameters such as the size of the break, the depth of the detachment (which is the distance between the posterior cornea and detached DM), the corneal thickness, and the varying degree of involvement of the PDL and DM [25,72,73]. The capture of these parameters upon initial presentation of acute hydrops might guide further management by corneal specialists, as it has been suggested that eyes with deeper DM detachment, larger breaks, and the presence of large intrastromal clefts have poorer prognosis and require a longer time for the resolution of corneal edema [72,74].

### 6.2. Corneal Dystrophy 

Corneal dystrophy refers to a group of corneal disease that are typically bilateral, symmetrical, slowly progressive, and independent of environmental and systemic factors [75]. It is now classified based on the IC3D classification, with dystrophies classified according to the affected layers, namely, epithelial, subepithelial, BM, stromal, DM, and endothelial dystrophies. These can also be grouped together based on known, common genetic bases [76].

Prior to the development of the slit lamp, the examination of the cornea was limited. Even with the slit lamp, the differential diagnosis of corneal dystrophies is challenging due to similarities in clinical phenotypes. The ability of AS-OCT to discriminate between the corneal layers in almost histological detail marks another potential breakthrough in the diagnosis of corneal dystrophies [77]. However, their precise diagnosis still requires significant clinical correlation.

### 6.3. Infectious and Noninfectious Keratitis

Another major application of AS-OCT in clinical practice is the diagnosis and management of keratitis. In keratitis, inflammatory cells infiltrate the different corneal layers in response to noxious stimuli. This is often associated with corneal edema and ciliary congestion and can lead to the suppurative melting of the corneal epithelium and stroma, with the formation of ulcers and the loss of corneal clarity. Corneal opacification is the fifth leading cause of blindness globally, with approximately 6 million people worldwide thought to suffer from cornea-related moderate or severe visual impairment [78,79]. The commonest form of keratitis is infectious keratitis (IK), which is the commonest cause for corneal blindness in both developed and developing countries [80,81,82]. Other noninfectious causes of keratitis include inflammation, autoimmune reactions, degeneration, and nutritional deficiencies. 

Traditionally, keratitis is diagnosed and monitored using a slit lamp by serially assessing the site and size of infiltrates and stromal ulceration [83]. However, evaluation of the depth and extent of pathologic features is subjective and depends significantly on the examiner’s experience, therefore limiting the reliability and consistency of the interpretation of disease progression and treatment response. This has been aided in recent years through the increased use of anterior segment serial photography, with a potential role for developing deep learning algorithms in the future [84,85].

AS-OCT has the potential to transform the way we manage IK by providing a more reliable and objective method of quantifying and monitoring the disease process [86]. Through the use of caliper tools provided by software, it enables the accurate determination and evaluation of the depth and extent of corneal ulceration, infiltrate (visualized as a hyper-reflective area in the stroma), and haze [5,25,87,88,89,90,91]. This is especially useful in scenarios when necrotic tissues or infiltrate obscure the view of the underlying tissues. The most commonly used measurements are the stromal infiltration thickness (IT) and the corneal thickness (CT). By adhering to the same scanning protocols, serial scans can be taken through the same affected corneal areas, allowing objective monitoring of the CT and IT throughout the disease course. In the initial stages of IK, even mild cases seem to have a thick CT in the infiltrated area, which decreases as the infection resolves, along with the IT. 

**Figure 9:**
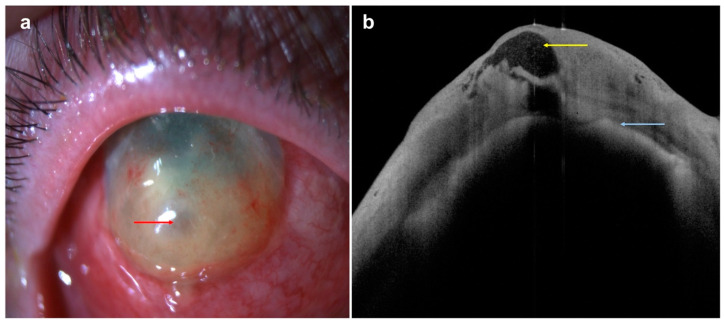
Severe *Pseudomonas aeruginosa* keratitis with corneal perforation: (**a**) slit-lamp photograph demonstrating significant corneal infiltrate and stromal melt/threatened perforation (red arrow), with a negative Seidel sign; (**b**) AS-OCT demonstrating significant corneal melt (yellow arrow) with a very thin residual layer of epithelium and 360-degree iridocorneal touch and absence of anterior chamber (blue arrow), indicating the presence of corneal perforation. The absence of anterior chamber resulted in a negative Seidel sign, providing a false reassurance of corneal integrity.

The infiltration can also be qualitatively assessed using AS-OCT, since the intensity of hyper-reflectivity corresponds to the density of the infiltration on slit lamp. However, it is important to interpret AS-OCT findings in the clinical context, as a decreasing CT might not always mean the resolution of infection. For example, if stromal necrosis occurs, such as with fungal keratitis and chronic bacterial keratitis, severe corneal thinning could be mistaken as an ‘improving’ CT. This is an ominous sign of impending corneal perforation, and this thinning can be detected on AS-OCT before it is apparent on slit-lamp examination (Figure 9). Furthermore, AS-OCT is also able to detect IT thickening before clinical deterioration, emphasizing that when used appropriately, AS-OCT can provide vital information on not only IK improvement but also on deterioration and the risk of corneal perforation [87]. 

**Figure 10:**
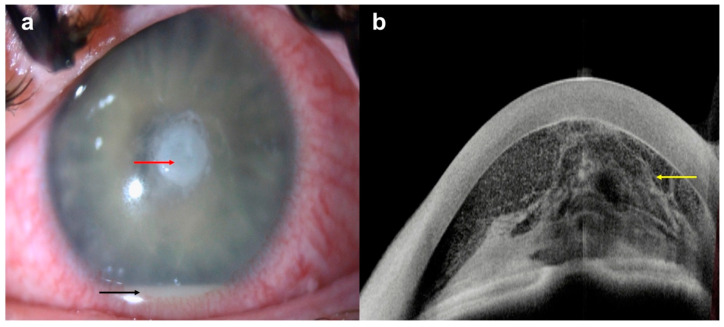
*Pseudomonas aeruginosa* keratitis with severe anterior chamber reaction: (**a**) slit-lamp photograph demonstrating a central infiltrate (red arrow) with severe anterior chamber reaction and hypopyon (black arrow); (**b**) AS-OCT demonstrating severe anterior chamber reaction, evidenced by increased hyper-reflective cellular changes in anterior chamber (yellow arrow).

**Figure 11:**
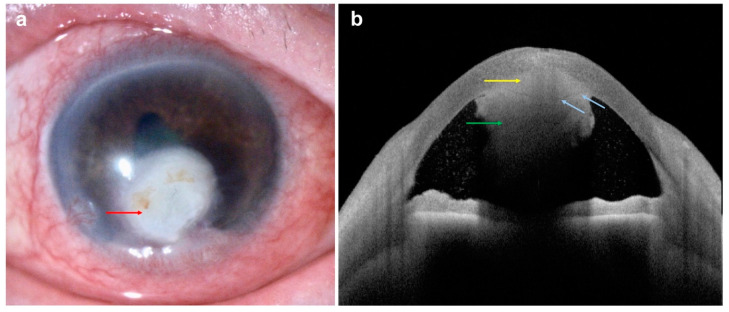
*Candida* keratitis with retrocorneal plaque: (**a**) slit-lamp photograph demonstrating a deep stromal infiltrate (red arrow) with extension into the anterior chamber; (**b**) AS-OCT demonstrating a posterior stromal infiltrate with a homogeneous increased hyper-reflectivity (yellow arrow) between posterior stroma and pre-Descemet layer/Descemet membrane (blue arrows), and hyper-reflectivity in anterior chamber (green arrow), suggesting an extension of fungal infection into the anterior chamber.

Additional features of IK that can be captured using AS-OCT include inflammatory cells/fibrin in the anterior chamber (AC), which appear as hyper-reflective cells (Figure 10), and retro-corneal pathology such as endothelial inflammatory plaques, typical of fungal keratitis [88,92,93] (Figure 11). The presence of these plaques often interferes with the accurate identification of the corneal layers and thus IT and CT measurement. However, by using AS-OCT, these plaques can be measured and monitored on serial scans as an alternative indicator of disease evolution. The extent of stromal edema, visualized as diffuse stromal thickening that changes the convexity of the posterior corneal surface, can also be assessed with AS-OCT, along with the presence of DM folds (ruffles in the usually smooth corneal endothelium) [87]. All this confirms that serial AS-OCT scans allow objective measurement of the disease and treatment response through both quantitative and qualitative information.

AS-OCT might also be of use in differentiating between different forms of IK [89,94]. For example, localized and diffuse stromal cystic spaces caused by stromal necrosis can be observed in fungal keratitis, along with endothelial plaques, as mentioned above [92,95]. In *Acanthamoeba* keratitis (AK), AS-OCT provides detailed visual images of radial keratoneuritis and perineural infiltrates, which appear as hyper-reflective bands in the corneal stroma of varying widths (20–200 μm) and depths (subepithelial to mid-stroma) [23,96,97]. AS-OCT also seems to be helpful in differentiating AK from herpetic keratitis, in which these hyper-reflective bands are predominantly observed in the subepithelial zone [97]. Furthermore, the common whitish, raised, epithelial lesions of ocular microsporidiosis can also be observed with AS-OCT as hyper-reflective epithelial lesions, rising above the epithelial surface. This aids in the differentiation from adenoviral nummular scars (hyper-reflective lesions starting from the subepithelial corneal layer and extending slightly into the anterior stroma, without elevations above the epithelium) [98,99]. Finally, in *Cytomegalovirus* (CMV) corneal endotheliitis, irregularly thickened hyper-reflective coin-shaped lesions have been observed at the endothelial surface, along with protruding hyper-reflective structures at the posterior cornea with a dendritic, dome-shaped, quadrangular, or saw-tooth appearance [100,101]. All these AS-OCT features could be used clinically to aid or confirm diagnosis and monitor response to treatment.

Another important infection that can affect the cornea is viral keratoconjunctivitis, a common, contagious infectious eye disease that is often caused by adenovirus. There are studies that have reported its prevalence to be up to 60% of patients presenting with a diagnosis of infectious keratoconjunctivitis [102,103]. One of its sequelae is the presence of nonstaining subepithelial infiltrates, which can persist even after the resolution of the acute episode [104]. Subepithelial infiltrates are thought to be due to immune hypersensitivity, which can then persist and form irreversible scars [105]. The treatment for this is usually a trial of topical steroids, with some evidence for the use of topical ciclosporin [106]. The use of AS-OCT in this clinical circumstance lies in its ability to discern subepithelial infiltrates that are potentially reversible [107].

### 6.4. Corneal Foreign Bodies

Corneal foreign bodies are a common type of ocular injury presenting with eye casualty. In the UK, it is estimated that there are 2.7 cases of corneal foreign body per 1000 population presenting to primary care [108]. In certain major industries such as car manufacturing, superficial corneal foreign body accounts for up to 70% of work-related injuries [109]. While most corneal foreign bodies are superficial and can be easily removed under a slit lamp, in certain cases, corneal foreign bodies can be deeply embedded and difficult to visualize due to corneal edema. The utility of AS-OCT in these cases would be to correctly identify the depth of the foreign body so that an appropriate removal method can be planned. Celebi et al. documented a case whereby AS-OCT demonstrated shadowing of the corneal layers, denoting the location of the corneal foreign body [110]. The corneal foreign body was subsequently removed externally as AS-OCT showed that it was not a full-thickness penetration. There are also studies that have shown that AS-OCT can provide clues about the distinguishing characteristics of foreign bodies, for example, opaque materials such as metal and wood tend to have a hyper-reflective anterior border, with posterior border signals blocked from the shadowing effect. With metal foreign bodies, there is also the appearance of a “mirror effect” with hyper-reflective signals either aligned or inverted with the direction of the foreign body, while transparent materials such as plastic and glass can have both hyper- and hyporeflective signals [111,112]. 

### 6.5. Epithelial Thickness Mapping in Corneal Disease

The corneal epithelium accounts for 1.03 D of the total corneal refractive power at the central 2 mm zone and 0.85 D of the total corneal refractive power over a 3.6 mm zone, with even the subtlest changes in epithelial thickness (ET) and morphology resulting in significant refractive changes [113]. The ET profile is nonuniform, with corneal epithelium being thicker inferiorly than superiorly and nasally than temporally, which is thought to be due to eyelid forces and blinking action on the superior cornea [114,115].

ET and stromal thicknesses (ST) have been measured with several devices, including high-frequency, scanning ultrasound biomicroscopy and in vivo confocal microscopy (IVCM) [116,117]. AS-OCT has been shown to provide good, repeatable, and reproducible corneal epithelial thickness mapping (ETM) in both normal and diseased corneas, through all map zones, with newer generations providing ETM over a larger zone [118,119]. The corneal ET maps can be subdivided into a total of seventeen zones: one central 2 mm diameter zone, eight 3 mm wide paracentral zones, and eight 1 mm wide outer peripheral zones. Through ETM, AS-OCT is also able to measure various other parameters such as minimum CT, minimum–median CT, inferior–superior ET difference, minimum–maximum ET difference, and standard deviation (SD) of the ET [120].

ETM, through the use of AS-OCT, has a wide range of clinical utility. As mentioned above, ETM can be used to diagnose early, subclinical keratoconus, which can be easily missed. The epithelium thins in the area over the cone and thickens around it (epithelial doughnut pattern), while as the disease progress, excessive epithelial thinning can occur [121]. Thus, ETM can be a valuable tool for monitoring progression alongside corneal tomography (such as Pentacam). Similarly, postectasia epithelial changes resemble those seen in keratoconus [120,122]. ETM can also help in differentiating contact-lens-induced warpage from keratoconus progression; as the former also leads to inferior steepening, it results in a thicker rather than thinner (as would happen with progressing keratoconus) epithelium [120].

ETM also has diagnostic and monitoring value in various ocular surface diseases. For example, in patients with dry eye disease as well as atopic disease, the ET decreases throughout the cornea, whereas in epithelial basement membrane dystrophy (EBMD), it thickens the central and inferior corneal areas [123,124,125]. Limbal stem cell deficiency (LSCD) is another corneal disorder that significantly affects the epithelium. In their study, Levy et al. found that ETM in LSCD was associated with a spoke-wheel pattern, a maximum to minimum ET difference greater than 14 μm, and an ET standard deviation greater than 5 μm [126].

The AS-OCT findings of these ocular surface diseases are also summarized in Table 2. 

## 7. Clinical Applications in Corneal and Refractive Surgeries

AS-OCT is playing an increasing role in the preoperative and intraoperative stages of cornea surgery due to its ability to discern the different layers, especially in the context of corneal transplantation [127]. The general ophthalmologists and eye care health professionals would most likely encounter postsurgical cornea patients in an urgent care setting, where AS-OCT can be used as an important diagnostic tool. 

### 7.1. Keratoplasty

Penetrating keratoplasty (PKP) was until recently the commonest type of corneal transplantation performed in the UK, though there has been a shift toward the less invasive partial thickness or lamellar keratoplasty techniques in recent years [128,129,130,131]. In fact, Descemet’s stripping automated endothelial keratoplasty (DSAEK) is nowadays considered to be the most common corneal transplantation technique in the UK, accounting for 33% of all cases of corneal transplant in the period of 2019–2020 [132]. Other lamellar keratoplasty techniques include deep anterior lamellar keratoplasty (DALK; 9.3% of all cases in 2019–2020) and Descemet membrane endothelial keratoplasty (DMEK; 23.1% of all cases in 2019–2020) [132].

PKP surgery involves the alignment of the host and donor cornea. AS-OCT provides details of the graft–host junction for both the anterior and posterior interface. In a study by Kaiserman et al. using AS-OCT to examine the wound configuration of patients after PKP, up to 60.8% of graft–host junctions had internal graft–host malappositions such as gapes, steps, or protrusions [133]. This is despite all the graft–host junction having a continuous smooth epithelial surface. They also observed a correlation between the extent of malapposition and degree of refractive error. Recently, AS-OCT has also been shown to recognize and characterize graft–host ectasia in post-PK eyes. It was found that a ratio of the lowest corneal thickness at the interface to central corneal thickness of ≤0.7 is associated with a significantly higher chance (2.4 times) of clinically detectable ectasia [134].

**Figure 12:**
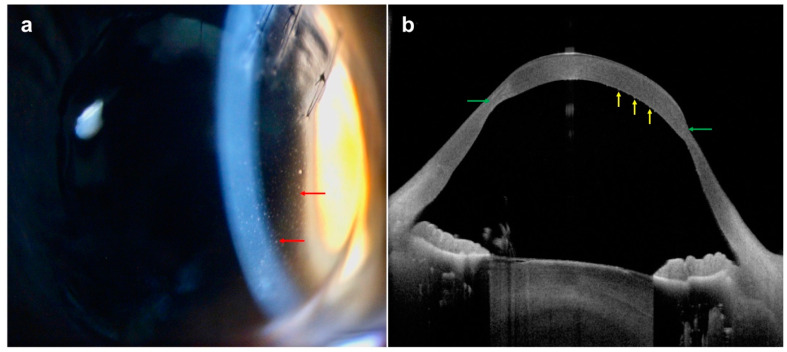
Corneal endothelial graft rejection: (**a**) slit-lamp photography demonstrating a mild corneal endothelial graft rejection with keratic precipitates (red arrows) in an eye with previous penetrating keratoplasty for keratoconus; (**b**) AS-OCT showing keratic precipitates as mild excrescences on the corneal endothelium (yellow arrows). Considerable graft–host ectasia (green arrows) is also noted.

The leading causes of corneal allograft failure are graft rejection and infection [135,136,137]. The clinical signs of corneal graft rejection may include conjunctival injection, epithelial rejection line, nummular subepithelial infiltrates, corneal edema, corneal vascularization, keratic precipitates, endothelial rejection line (Khodadoust line), and/or anterior chamber inflammatory activity [138]. With the use of AS-OCT, corneal edema can be monitored objectively using its pachymetry tool. In addition, subepithelial infiltrates, stromal thickening, and keratic precipitates can be directly visualized (Figure 12). There are suggestions that acute graft rejection can present on AS-OCT as stromal thickening and posterior corneal surface undulation [139]. Following treatment with topical and systemic steroids, the stromal thickness decreases, with the corresponding resolution of the posterior undulations. In contrast, patients with chronic graft failure are likely to have persistent stromal thickening refractory to steroid therapy, with a smooth posterior corneal surface. 

DALK is a form of lamellar keratoplasty that selectively replaces the anterior part of the cornea, sparing the healthy DM and endothelium. This technique relies on the separation of the stroma from the DM, with the big bubble technique developed by Anwar and Teichmann being one of the more popular approaches [140]. Following DALK surgery, AS-OCT can provide high-resolution imaging of the graft–host junction as well as the graft–host interface, enabling the assessment of complications such as DM nonattachment. DM nonattachment following DALK (similar to DM detachment) can be difficult to identify accurately on slit lamp due to epithelial and stromal swelling. Advantageously, AS-OCT can provide detailed information such as the configuration of the nonattachment, as well as whether it is local (less than 1/3 of the corneal surface) or extensive (more than 1/3 of the cornea surface) [141]. 

**Figure 13:**
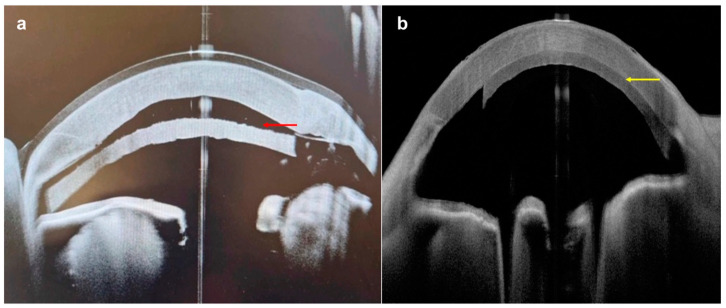
Descemet stripping automated endothelial keratoplasty in an aphakic eye with previous glaucoma tube drainage surgery: (**a**) AS-OCT demonstrating complete graft detachment postoperatively (red arrow); (**b**) complete graft attachment was achieved (yellow arrow) after rebubbling and suturing of the glaucoma tube.

On the other hand, DSAEK and DMEK are posterior lamellar keratoplasty techniques in which the recipient’s diseased DM and endothelium are replaced. In traditional DSAEK, diseased endothelium is replaced with donor tissue comprising a thin layer of posterior stroma, DM, and endothelium. The DSAEK tissue is usually around 150–200 µm, although ultrathin DSAEK can be as thin as ~80 µm [142]. Comparatively, DMEK is a newer technique that only transplants the DM and endothelium and is only about 10–15 µm in thickness [143,144,145,146]. In both DSAEK and DMEK, one of the potential complications is graft detachment [147,148,149,150,151]. Similar to what we described for DALK above, the status of the donor and host interface has been historically monitored using slit-lamp examination, which can be challenging in the immediate postoperative period due to corneal edema from chronical endothelial failure. The benefit of AS-OCT is that it can visualize the donor–host interface and provide information on donor apposition and the extent of DM detachment, thereby guiding the need and techniques for rebubbling (if indicated) [149,152] (Figure 13). Studies have shown that AS-OCT performed at 1 h and 1 week after DMEK surgery was able to facilitate the visualization of the extent of DMEK graft attachment and the decision making on rebubbling of the graft [153]. For instance, the presence of large graft detachment (>33% of the total graft surface) 1 h postoperatively is associated with a 75% chance of persistent large or complete graft detachment at 6 months.

**Figure 14:**
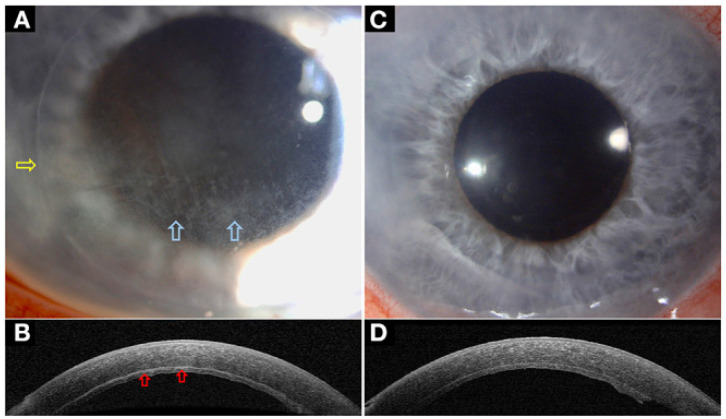
A case of interface infectious keratitis (IIK) following Descemet stripping automated endothelial keratoplasty (DSAEK): (**A**) slit-lamp photography demonstrating an inflamed right eye with diffused stromal haze in a crisscross pattern at the graft–host interface (blue arrows), consistent with a diagnosis of IIK. The edge of the DSAEK graft is visible (yellow arrow); (**B**) hyper-reflective changes at the graft–host interface (red arrows) are clearly delineated on AS-OCT, highlighting the value of AS-OCT in facilitating the assessment of infectious keratitis; (**C**) slit-lamp photography demonstrating complete resolution of the IIK following intensive topical antifungal treatment, evidenced by the disappearance of the stromal haze on slit-lamp photograph; (**D**) this corresponds to the disappearance of the hyper-reflective changes at the graft–host interface on AS-OCT (this figure was adapted from Song et al. [155] and was reproduced unchanged under the terms of the CC BY 4.0 license, https://creativecommons.org/licenses/by/4.0/, accessed on 5 November 2023).

AS-OCT can also be employed to visualize and monitor the progression of interface infectious keratitis (Figure 14), which is a distinct and challenging clinical entity following lamellar keratoplasty [154,155,156].

### 7.2. Refractive Surgery

According to a refractive surgery market report, the global demand for refractive surgical procedures is estimated as growing at a compound rate of 9.6% from 2020 to 2025, with the number of procedures increasing from 3.6 million to 5.8 million [157]. 

Laser in situ keratomileusis (LASIK) remains one of the most commonly performed corneal refractive procedures [158,159]. The LASIK technique involves the creation of a ‘hinged’ flap before performing excimer laser ablation on the corneal stromal bed [160,161]. Although LASIK has a well-documented safety profile, one of the complications is interface fluid syndrome, which is the presence of liquid at the LASIK flap interface [162]. This can be associated with steroid-induced raised intraocular pressure, endothelial decompensation, and other pathophysiologic processes, which result in cornea edema and can occur weeks and months after LASIK procedures [162]. Clinically, it can be challenging to diagnose due to its appearance as diffuse corneal haze, which can mimic diffuse lamellar keratitis [163]. AS-OCT confers the advantage of confirming the diagnosis of interface fluid syndrome by demonstrating hyporeflective fluid at the flap interface [9,164]. 

**Figure 15:**
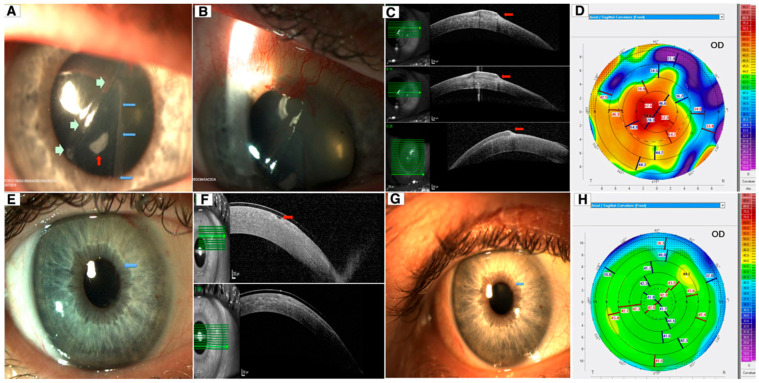
Late-onset traumatic dislocation of LASIK flap: (**A**,**B**) slit-lamp photos revealing a right hazy and thickened corneal flap, central epithelial ingrowth (red arrow), superior-nasal corneal neovascularization, and a large area of nasal corneal flap folded and tucked under the central flap (green arrows—edge of the original nasal flap; blue arrows—edge of the fold); (**C**) AS-OCT delineating the extent of the infolded flap (red arrows); (**D**) Scheimpflug corneal tomography showing significant corneal flattening nasally with irregular astigmatism and steeping centrally; (**E**) slit-lamp photo showing a clear cornea with an area of very mild interface haze (blue arrow) following the repositioning of the dislocated LASIK flap and removal of epithelial ingrowth; (**F**) AS-OCT demonstrating a small hole within the LASIK flap postoperatively (red arrow); (**G**) mild recurrence of epithelial ingrowth (blue, left arrow) was noted 9 months postoperatively; (**H**) corneal tomography showing significant improvement in the anterior corneal curvature and irregular astigmatism 9 months post-operation (this figure was adapted from Ting et al. [91] with permission from the licensor Springer Nature, https://link.springer.com/article/10.1007/s10792-018-0946-7#Fig1, accessed on 14 November 2023).

Another potential complication of LASIK is flap displacement/dislocation. This can be easily missed on slit-lamp examination due to the difficulty in identifying the flap edge. There have been several case reports that have demonstrated the superiority of AS-OCT in detecting flap gaps [91,165]. Ting and Danjoux reported three cases of late-onset (7–14 years postoperatively) traumatic dislocation of LASIK flaps, all with delayed diagnosis and management [91]. All three had signs detected on AS-OCT: two with an infolded flap and one with epithelial ingrowth. AS-OCT can also be utilized to identify and monitor the progression and treatment response for epithelial ingrowth [166] (Figure 15).

**Figure 16:**
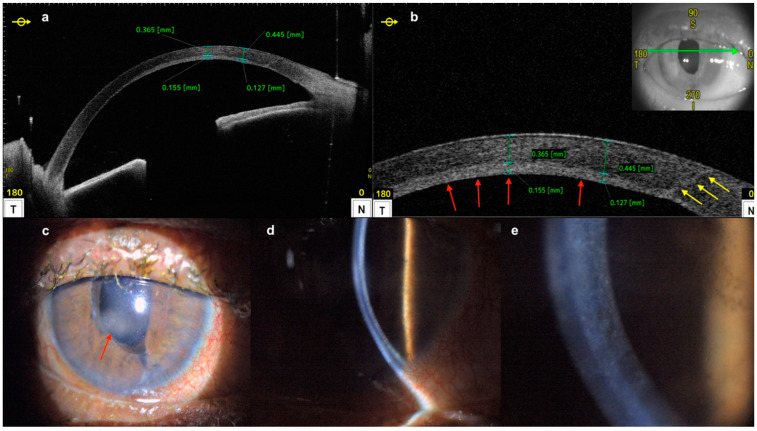
Epithelial ingrowth postcataract surgery: (**a**,**b**) AS-OCT showing a hyper-reflective layer of presumed epithelial cells (red arrows) extending through the previous paracentesis (yellow arrows); (**c**–**e**) slit-lamp images showing the corresponding area of epithelial ingrowth.

Epithelial ingrowth (also termed epithelial downgrowth) is a rare but sight-threatening complication that can also occur after other ocular surgery techniques, including cataract surgery (Figure 16), clear lens exchange, and PKP [167,168,169,170]. It is characterized by a translucent sheet of epithelial cells that spreads across anterior chamber tissues. Its incidence is reported to range from 0.08 to 0.12% after extracapsular or intracapsular cataract surgery and around 0.27% after PKP [169,170]. It can lead to intractable intraocular inflammation, high intraocular pressure, and glaucoma.

### 7.3. Epithelial Thickness Mapping and Refractive Surgery

ETM has been increasingly used during preoperative planning of refractive surgery [120]. A major issue after refractive surgery is iatrogenic post-LASIK ectasia (PLE), with preoperative subclinical early-stage keratoconus thought to play a major role. PLE is a serious complication characterized by a gradual loss of vision, localized steepening distortion and thinning of the cornea, and progressive irregular astigmatism [171,172]. Even though nowadays corneal topography is still considered to be the gold standard for corneal ectasia, there have been reports of eyes with seemingly normal topography and no other known risk factors proceeding to develop PLE [173,174,175]. 

In refractive surgery, it is essential that the therapeutic profile applied is based on the real shape of the stromal surface in a true stromal surface topography-guided ablation [176]. The corneal epithelium is known to remodel as a result of underlying stromal irregularities and helps mask them [177,178]. As mentioned above, the epithelium is not evenly distributed over the BL and attempts to smoothen out abrupt changes in the stromal surface by thinning focally over; hills’ and thickening locally over the ‘valleys’ of an irregular stroma [179]. Due to this epithelial masking, topography and wavefront measurements may not provide an accurate picture of the underlying irregular stromal surface, leading to suboptimal outcomes as they only correct for the proportion of the stromal irregularity that is not masked by the epithelium [176]. This emphasizes the need for precise, preoperative, independent imaging and analysis of the corneal epithelium and stroma via AS-OCT. Accurate preoperative analysis of the ET distribution pattern helps guide whether to defer refractive surgery in subclinical cases with suspicious topography, as well as providing the opportunity for appropriate treatment in the form of corneal crosslinking [180].

When planning transepithelial photorefractive keratectomy (trans-PRK) and LASIK flaps, customization of the ablation depth (for trans-PRK) and flap thickness (for LASIK) based on ETM is essential, as undercorrection due to lesser stromal ablation with standard, noncustomized treatment may occur [120]. Accurate ET measurement is also important in planning phototherapeutic keratectomy (PTK), which aims to treat opacities and irregularities of the corneal surface using an excimer laser. With accurate ETM, transepithelial PTK can reach the required depth of epithelium and anterior stroma using a stepwise protocol, improving the regularity of the stromal surface, along with the visual acuity [181]. 

Apart from pre-operative planning of refractive surgery treatment protocols, ETM also has an important role in evaluating post-operative outcomes, since irregular epithelial remodeling post-refractive surgery can lead to regression. Accurate and repeatable measurements of the ET help improve earlier detection of postoperative iatrogenic ectasia [181]. Furthermore, it also helps in the planning of recorrection, improving safety and efficacy [182].

## 8. Conclusions

The advances and commercialization of AS-OCT since its first introduction in 1994 have led to increased clinical applications, and it is likely to change the way ophthalmologists diagnose and manage anterior segment conditions. While we did not cover this in our review, AS-OCT also has significant applications in glaucoma due to its ability to image the anterior segment angle [183]. The rich datasets produced by AS-OCT will likely pave the way for future applications with artificial intelligence as a clinical diagnostic aid [184]. Despite the numerous advantages of AS-OCT over slit lamp in certain contexts, the slit lamp will likely continue remaining the default diagnostic equipment due to its ubiquity and versatility in the examination of both the anterior and posterior structures of the eye. However, AS-OCT is increasingly becoming an indispensable tool within the diagnostic armamentarium of the modern ophthalmic examination. 

## Figures and Tables

**Table 2:** AS-OCT features of various ocular surface diseases.

Disease	AS-OCT Features
Conjunctival melanocytic naevus	Detection of intralesional cysts [29,30].
Ocular surface squamous neoplasia (OSSN)	Abrupt transition of normal conjunctival epithelium to abnormal epithelium, characterized by thicker hyper-reflective lesions [33].
Conjunctival melanoma	Normal basal epithelium, with abnormal subepithelial hyper-reflectivity representing atypical melanocytes [36]. UHR AS-OCT should be used to improve penetration through thick pigmented lesions [36].
Conjunctival lymphoma	Homogenous, dark subepithelial lesion with smooth borders, containing monomorphic dot-like infiltrates [38].
Pterygium	Subepithelial hyper-reflective lesion from the conjunctiva with extension into the cornea, with separation of the cornea epithelium from BM [48].
Pinguecula	Subepithelial hyper-reflective lesion from the conjunctiva without extension into the cornea (stops short at the corneal limbus).
Salzmann nodular degeneration	Hyper-reflective lesion on the cornea limited to the subepithelial corneal layer.
Pseudopterygium	Overgrowing membrane that is not attached to the underlying cornea [48].
Episcleritis	Increased episcleral and episcleral network thickness, with minimal changes in scleral thickness [52].
Scleritis	Increase in hyporeflective spaces at variable depths, corresponding to dilated superficial and deep episcleral blood vessels [53]. Total combined thickness of the conjunctiva, episcleral and sclera can be increased.
Keratoconus	Thinner-than-average cornea with focal thinning inferiorly, demonstrated on quantitative pachymetry maps and epithelial thickness mapping [61,63,65].
Acute corneal hydrops	Integrity of the PDL/DM and the break can be visualized and characterized, based on parameters such as the size of the break, the depth of the detachment (which is the distance between the posterior cornea and detached DM), the corneal thickness, and the varying degree of involvement of PDL and DM [25,72,73].
Infectious keratitis	Accurate determination and evaluation of the depth and extent of corneal ulceration, infiltrate (visualized as a hyper-reflective area in the stroma), and haze [5,25,87,88,89,90,91]. Qualitative assessment of infiltrate: intensity of hyper-reflectivity of infiltrate corresponds to the density of the infiltration on slit lamp; infiltrate thickening picked up before clinical deterioration [87]. Improving infection with improving infiltrate thickness and corneal thickness. Impending corneal perforation with ‘improving’ corneal thickness [87]. Hyper-reflective inflammatory cells/fibrin in the anterior chamber Stromal edema, visualized as diffuse stromal thickening that changes the convexity of the posterior corneal surface, along with the presence of DM folds (ruffles in the usually smooth corneal endothelium) [87]. Differentiation of different form of infectious keratitis: Fungal keratitis—localized and diffuse stromal cystic spaces caused by stromal necrosis, along with endothelial inflammatory plaques [92,95]. *Acanthamoeba* keratitis—radial keratoneuritis and perineural infiltrates, which appear as hyper-reflective bands in the corneal stroma of varying widths (20–200 μm) and depths (subepithelial to midstroma) [23,96,97]. Herpetic keratitis—hyper-reflective bands predominantly observed in the subepithelial zone [97]. Ocular microsporidiosis—hyper-reflective epithelial lesions, rising above the epithelial surface. Adenoviral keratoconjunctivitis—hyper-reflective lesions starting from the subepithelial corneal layer and extending slightly into the anterior stroma, without elevations above the epithelium [98,99]. *Cytomegalovirus* corneal endotheliitis—irregularly thickened hyper-reflective coin-shaped lesions at the endothelial surface, along with protruding hyper-reflective structures at the posterior cornea with a dendritic, dome-shaped, quadrangular, or saw-tooth appearance [100,101].
Corneal foreign body	Shadowing of the corneal layers denoting the location of the corneal foreign body [110]. Clues about the distinguishing characteristics of foreign bodies, for example, opaque materials such as metal and wood tend to have a hyper-reflective anterior border, with posterior border signals blocked from shadowing effect. With metal foreign bodies, there is also the appearance of a ‘mirror effect’ with hyper-reflective signals either aligned or inverted with the direction of the foreign body, whereas transparent materials such as plastic and glass can have both hyper- and hyporeflective signals [111,112].

## Data Availability

This review produced no new research data.

## References

[1] Gabriele M.L., Wollstein G., Ishikawa H., Kagemann L., Xu J., Folio L.S., Schuman J.S. (2011). Optical Coherence Tomography: History, Current Status, and Laboratory Work. Investig. Ophthalmol. Vis. Sci..

[2] Chopra R., Wagner S.K., Keane P.A. (2021). Optical Coherence Tomography in the 2020s—Outside the Eye Clinic. Eye.

[3] Müller P.L., Wolf S., Dolz-Marco R., Tafreshi A., Schmitz-Valckenberg S., Holz F.G. (2019). Ophthalmic Diagnostic Imaging: Retina. High Resolution Imaging in Microscopy and Ophthalmology: New Frontiers in Biomedical Optics.

[4] Izatt J.A., Hee M.R., Swanson E.A., Lin C.P., Huang D., Schuman J.S., Puliafito C.A., Fujimoto J.G. (1994). Micrometer-Scale Resolution Imaging of the Anterior Eye In Vivo with Optical Coherence Tomography. Arch. Ophthalmol..

[5] Ang M., Baskaran M., Werkmeister R.M., Chua J., Schmidl D., Dos Santos V.A., Garhöfer G., Mehta J.S., Schmetterer L. (2018). Anterior Segment Optical Coherence Tomography. Prog. Retin. Eye Res..

[6] Triolo G., Barboni P., Savini G., De Gaetano F., Monaco G., David A., Scialdone A. (2021). The Use of Anterior-Segment Optical-Coherence Tomography for the Assessment of the Iridocorneal Angle and Its Alterations: Update and Current Evidence. J. Clin. Med..

[7] Lee W.D., Devarajan K., Chua J., Schmetterer L., Mehta J.S., Ang M. (2019). Optical Coherence Tomography Angiography for the Anterior Segment. Eye Vis..

[8] Alió J.L., Del Barrio J.L.A. (2021). Atlas of Anterior Segment Optical Coherence Tomography.

[9] Ramos J.L.B., Li Y., Huang D. (2009). Clinical and Research Applications of Anterior Segment Optical Coherence Tomography—A Review. Clin. Exp. Ophthalmol..

[10] Drexler W., Liu M., Kumar A., Kamali T., Unterhuber A., Leitgeb R.A. (2014). Optical Coherence Tomography Today: Speed, Contrast, and Multimodality. J. Biomed. Opt..

[11] Keane P.A., Ruiz-Garcia H., Sadda S.R. (2011). Clinical Applications of Long-Wavelength (1000-Nm) Optical Coherence Tomography. Ophthalmic Surg. Lasers Imaging.

[12] Venkateswaran N., Mercado C., Wall S.C., Galor A., Wang J., Karp C.L. (2021). High Resolution Anterior Segment Optical Coherence Tomography of Ocular Surface Lesions: A Review and Handbook. Expert. Rev. Ophthalmol..

[13] Drexler W., Morgner U., Ghanta R.K., Kärtner F.X., Schuman J.S., Fujimoto J.G. (2001). Ultrahigh-Resolution Ophthalmic Optical Coherence Tomography. Nat. Med..

[14] Aumann S., Donner S., Fischer J., Müller F. (2019). Optical Coherence Tomography (OCT): Principle and Technical Realization. High Resolution Imaging in Microscopy and Ophthalmology: New Frontiers in Biomedical Optics.

[15] Bille J.F. (2019). High Resolution Imaging in Microscopy and Ophthalmology: New Frontiers in Biomedical Optics.

[16] Optopol Technology (2014). SOCT Copernicus HR.

[17] Bald M., Li Y., Huang D. (2012). Anterior Chamber Angle Evaluation with Fourier-Domain Optical Coherence Tomography. J. Ophthalmol..

[18] Radhakrishnan S., Rollins A.M., Roth J.E., Yazdanfar S., Westphal V., Bardenstein D.S., Izatt J.A. (2001). Real-Time Optical Coherence Tomography of the Anterior Segment at 1310 Nm. Arch. Ophthalmol..

[19] Rio-Cristobal A., Martin R. (2014). Corneal Assessment Technologies: Current Status. Surv. Ophthalmol..

[20] Topcon Healthcare (2022). DRI OCT Triton Series—A Multimodal Swept Source OCT.

[21] Optovue Inc (2022). Optovue Iseries.

[22] Dua H.S., Darren S.J., Al-Aqaba T.M., Said D.G. (2023). Pathophysiology of Keratoconus. Keratoconus.

[23] Azzopardi M., Chong Y.J., Ng B., Recchioni A., Logeswaran A., Ting D.S.J. (2023). Diagnosis of Acanthamoeba Keratitis: Past, Present and Future. Diagnostics.

[24] Werkmeister R.M., Alex A., Kaya S., Unterhuber A., Hofer B., Riedl J., Bronhagl M., Vietauer M., Schmidl D., Schmoll T. (2013). Measurement of Tear Film Thickness Using Ultrahigh-Resolution Optical Coherence Tomography. Investig. Ophthalmol. Vis. Sci..

[25] Dua H.S., Sinha R., D’Souza S., Potgieter F., Ross A., Kenawy M., Scott I., Said D.G. (2020). “Descemet Membrane Detachment”: A Novel Concept in Diagnosis and Classification. Am. J. Ophthalmol..

[26] Shields C.L., Demirci H., Karatza E., Shields J.A. (2004). Clinical Survey of 1643 Melanocytic and Nonmelanocytic Conjunctival Tumors. Ophthalmology.

[27] Shields C.L., Fasiudden A., Mashayekhi A., Shields J.A. (2004). Conjunctival Nevi: Clinical Features and Natural Course in 410 Consecutive Patients. Arch. Ophthalmol..

[28] Shields J.A., Shields C.L. (2008). Eyelid, Conjunctival and Orbital Tumors. An Atlas and Textbook.

[29] Vizvári E., Skribek Á., Polgár N., Vörös A., Sziklai P., Tóth-Molnár E. (2018). Conjunctival Melanocytic Naevus: Diagnostic Value of Anterior Segment Optical Coherence Tomography and Ultrasound Biomicroscopy. PLoS ONE.

[30] Shields C.L., Belinsky I., Romanelli-Gobbi M., Guzman J.M., Mazzuca D., Green W.R., Bianciotto C., Shields J.A. (2011). Anterior Segment Optical Coherence Tomography of Conjunctival Nevus. Ophthalmology.

[31] Höllhumer R., Williams S., Michelow P. (2021). Ocular Surface Squamous Neoplasia: Management and Outcomes. Eye.

[32] Shields J.A., Shields C.L., Scartozzi R. (2004). Survey of 1264 Patients with Orbital Tumors and Simulating Lesions: The 2002 Montgomery Lecture, Part 1. Ophthalmology.

[33] Ong S.S., Vora G.K., Gupta P.K. (2016). Anterior Segment Imaging in Ocular Surface Squamous Neoplasia. J. Ophthalmol..

[34] Kenawy N., Lake S.L., Coupland S.E., Damato B.E. (2013). Conjunctival Melanoma and Melanocytic Intra-Epithelial Neoplasia. Eye.

[35] Wong J.R., Nanji A.A., Galor A., Karp C.L. (2014). Management of Conjunctival Malignant Melanoma: A Review and Update. Expert. Rev. Ophthalmol..

[36] Shousha M.A., Karp C.L., Canto A.P., Hodson K., Oellers P., Kao A.A., Bielory B., Matthews J., Dubovy S.R., Perez V.L. (2013). Diagnosis of Ocular Surface Lesions Using Ultra-High-Resolution Optical Coherence Tomography. Ophthalmology.

[37] Shields C.L., Chien J.L., Surakiatchanukul T., Sioufi K., Lally S.E., Shields J.A. (2017). Conjunctival Tumors: Review of Clinical Features, Risks, Biomarkers, and Outcomes—The 2017 J. Donald M. Gass Lecture. Asia Pac. J. Ophthalmol..

[38] Venkateswaran N., Mercado C., Tran A.Q., Garcia A., Diaz P.F.M., Dubovy S.R., Galor A., Karp C.L. (2019). The Use of High Resolution Anterior Segment Optical Coherence Tomography for the Characterization of Conjunctival Lymphoma, Conjunctival Amyloidosis and Benign Reactive Lymphoid Hyperplasia. Eye Vis..

[39] Venkateswaran N., Galor A., Wang J., Karp C.L. (2018). Optical Coherence Tomography for Ocular Surface and Corneal Diseases: A Review. Eye Vis..

[40] Bradley J.C., Yang W., Bradley R.H., Reid T.W., Schwab I.R. (2010). The Science of Pterygia. Br. J. Ophthalmol..

[41] Ting D.S.J., Liu Y.-C., Patil M., Ji A.J.S., Fang X.L., Tham Y.C., Lee Y.-F., Htoon H.M., Mehta J.S. (2021). Proposal and Validation of a New Grading System for Pterygium (SLIT2). Br. J. Ophthalmol..

[42] Liu L., Wu J., Geng J., Yuan Z., Huang D. (2013). Geographical Prevalence and Risk Factors for Pterygium: A Systematic Review and Meta-Analysis. BMJ Open.

[43] Forrester J.V., Dick A.D., McMenamin P.G., Roberts F., Pearlman E. (2020). The Eye E-Book: Basic Sciences in Practice.

[44] Norn M.S. (1979). Prevalence of Pinguecula in Greenland and in Copenhagen, and Its Relation to Pterygium and Spheroid Degeneration. Acta Ophthalmol..

[45] Panchapakesan J., Hourihan F., Mitchell P. (1998). Prevalence of Pterygium and Pinguecula: The Blue Mountains Eye Study. Aust. N. Z. J. Ophthalmol..

[46] Fotouhi A., Hashemi H., Khabazkhoob M., Mohammad K. (2009). Prevalence and Risk Factors of Pterygium and Pinguecula: The Tehran Eye Study. Eye.

[47] Viso E., Gude F., Rodríguez-Ares M.T. (2011). Prevalence of Pinguecula and Pterygium in a General Population in Spain. Eye.

[48] Soliman W., Mohamed T.A. (2012). Spectral Domain Anterior Segment Optical Coherence Tomography Assessment of Pterygium and Pinguecula. Acta Ophthalmol..

[49] Urbinati F., Borroni D., Rodríguez-Calvo-de-Mora M., Sánchez-González J.-M., García-Lorente M., Zamorano-Martín F., Rachwani-Anil R., Ortiz-Pérez S., Romano V., Rocha-de-Lossada C. (2022). Pseudopterygium: An Algorithm Approach Based on the Current Evidence. Diagnostics.

[50] Whitcup S.M., Sen H.N. (2021). Whitcup and Nussenblatt’s Uveitis: Fundamentals and Clinical Practice.

[51] Okhravi N., Odufuwa B., McCluskey P., Lightman S. (2005). Scleritis. Surv. Ophthalmol..

[52] Preetam Peraka R., Murthy S.I. (2023). Role of Anterior Segment Optical Coherence Tomography in Scleral Diseases: A Review. Semin. Ophthalmol..

[53] Levison A.L., Lowder C.Y., Baynes K.M., Kaiser P.K., Srivastava S.K. (2016). Anterior Segment Spectral Domain Optical Coherence Tomography Imaging of Patients with Anterior Scleritis. Int. Ophthalmol..

[54] McGhee C.N. (2013). Keratoconus: The arc of past, present and future. Clin. Exp. Optom..

[55] Santodomingo-Rubido J., Carracedo G., Suzaki A., Villa-Collar C., Vincent S.J., Wolffsohn J.S. (2022). Keratoconus: An Updated Review. Cont. Lens Anterior Eye.

[56] Cresta F.B., Orlandin L.F., Lucas M.B., Almodin E., Nassaralla B.A., Sandes J. (2022). Clinical Presentation and Evolution of Keratoconus. Keratoconus: A Comprehensive Guide to Diagnosis and Treatment.

[57] Pérez J.F., Marcos A.V., Peña F.J.M. (2014). Early Diagnosis of Keratoconus: What Difference Is It Making?. Br. J. Ophthalmol..

[58] Ting D.S.J., Rana-Rahman R., Chen Y., Bell D., Danjoux J.-P., Morgan S.J., Ghosh S., Baylis O. (2019). Effectiveness and Safety of Accelerated (9 mW/cm^2^) Corneal Collagen Cross-Linking for Progressive Keratoconus: A 24-Month Follow-Up. Eye.

[59] Deshmukh R., Ong Z.Z., Rampat R., Alió Del Barrio J.L., Barua A., Ang M., Mehta J.S., Said D.G., Dua H.S., Ambrósio R. (2023). Management of Keratoconus: An Updated Review. Front. Med..

[60] Wu D., Lim D.K.-A., Lim B.X.H., Wong N., Hafezi F., Manotosh R., Lim C.H.L. (2021). Corneal Cross-Linking: The Evolution of Treatment for Corneal Diseases. Front. Pharmacol..

[61] Li Y., Meisler D.M., Tang M., Lu A.T.H., Thakrar V., Reiser B.J., Huang D. (2008). Keratoconus Diagnosis with Optical Coherence Tomography Pachymetry Mapping. Ophthalmology.

[62] Kim J.S., Rho C.R., Cho Y.W., Shin J. (2021). Comparison of Corneal Thickness Measurements Using Ultrasound Pachymetry, Noncontact Tonopachy, Pentacam HR, and Fourier-Domain OCT. Medicine.

[63] Yücekul B., Dick H.B., Taneri S. (2022). Systematic Detection of Keratoconus in OCT: Corneal and Epithelial Thickness Maps. J. Cataract. Refract. Surg..

[64] Herber R., Lenk J., Pillunat L.E., Raiskup F. (2022). Comparison of Corneal Tomography Using a Novel Swept-Source Optical Coherence Tomographer and Rotating Scheimpflug System in Normal and Keratoconus Eyes: Repeatability and Agreement Analysis. Eye Vis..

[65] Yang Y., Pavlatos E., Chamberlain W., Huang D., Li Y. (2021). Keratoconus Detection Using OCT Corneal and Epithelial Thickness Map Parameters and Patterns. J. Cataract. Refract. Surg..

[66] Wolter J.R., Henderson J.W., Clahassey E.G. (1967). Ruptures of Descemet’s Membrane in Keratoconus Causing Acute Hydrops and Posterior Keratoconus. Am. J. Ophthalmol..

[67] Shaw E.L. (1976). Pathophysiology and Treatment of Corneal Hydrops. Ophthalmic Surg..

[68] Ting D.S.J., Srinivasan S. (2014). Pneumodescemetopexy with Perfluoroethane (C2F6) for the Treatment of Acute Hydrops Secondary to Keratoconus. Eye.

[69] Ting D.S.J., Said D.G., Dua H.S. (2019). Are Descemet Membrane Ruptures the Root Cause of Corneal Hydrops in Keratoconic Eyes?. Am. J. Ophthalmol..

[70] Fan Gaskin J.C., Patel D.V., McGhee C.N.J. (2014). Acute Corneal Hydrops in Keratoconus—New Perspectives. Am. J. Ophthalmol..

[71] Kreps E.O., Claerhout I., Koppen C. (2019). The Outcome of Scleral Lens Fitting for Keratoconus with Resolved Corneal Hydrops. Cornea.

[72] Basu S., Vaddavalli P.K., Vemuganti G.K., Ali M.H., Murthy S.I. (2012). Anterior Segment Optical Coherence Tomography Features of Acute Corneal Hydrops. Cornea.

[73] Dua H.S., Freitas R., Mohammed I., Ting D.S., Said D.G. (2023). The Pre-Descemet’s Layer (Dua’s Layer, Also Known as the Dua-Fine Layer and the Pre-Posterior Limiting Lamina Layer): Discovery, Characterisation, Clinical and Surgical Applications, and the Controversy. Prog. Retin. Eye Res..

[74] Gokul A., Krishnan T., Emanuel P.O., Saunders M., McGhee C.N. (2015). Persisting Extreme Acute Corneal Hydrops with a Giant Intrastromal Cleft Secondary to Keratoconus. Clin. Exp. Optom..

[75] Soh Y.Q., Kocaba V., Weiss J.S., Jurkunas U.V., Kinoshita S., Aldave A.J., Mehta J.S. (2020). Corneal Dystrophies. Nat. Rev. Dis. Primers.

[76] Weiss J.S., Møller H.U., Lisch W., Kinoshita S., Aldave A.J., Belin M.W., Kivelä T., Busin M., Munier F.L., Seitz B. (2008). The IC3D Classification of the Corneal Dystrophies. Cornea.

[77] Siebelmann S., Scholz P., Sonnenschein S., Bachmann B., Matthaei M., Cursiefen C., Heindl L.M. (2018). Anterior Segment Optical Coherence Tomography for the Diagnosis of Corneal Dystrophies According to the IC3D Classification. Surv. Ophthalmol..

[78] Flaxman S.R., Bourne R.R., Resnikoff S., Ackland P., Braithwaite T., Cicinelli M.V., Das A., Jonas J.B., Keeffe J., Kempen J.H. (2017). Global Causes of Blindness and Distance Vision Impairment 1990–2020: A Systematic Review and Meta-Analysis. Lancet Glob. Health.

[79] Ting D.S.J., Ho C.S., Deshmukh R., Said D.G., Dua H.S. (2021). Infectious Keratitis: An Update on Epidemiology, Causative Microorganisms, Risk Factors, and Antimicrobial Resistance. Eye.

[80] Ung L., Bispo P.J., Shanbhag S.S., Gilmore M.S., Chodosh J. (2019). The Persistent Dilemma of Microbial Keratitis: Global Burden, Diagnosis, and Antimicrobial Resistance. Surv. Ophthalmol..

[81] Stapleton F. (2023). The Epidemiology of Infectious Keratitis. Ocul. Surf..

[82] Ting D.S.J., Ho C.S., Cairns J., Elsahn A., Al-Aqaba M., Boswell T., Said D.G., Dua H.S. (2021). 12-Year Analysis of Incidence, Microbiological Profiles and In Vitro Antimicrobial Susceptibility of Infectious Keratitis: The Nottingham Infectious Keratitis Study. Br. J. Ophthalmol..

[83] Ting D.S.J., Cairns J., Gopal B.P., Ho C.S., Krstic L., Elsahn A., Lister M., Said D.G., Dua H.S. (2021). Risk Factors, Clinical Outcomes, and Prognostic Factors of Bacterial Keratitis: The Nottingham Infectious Keratitis Study. Front. Med..

[84] Li Z., Jiang J., Chen K., Chen Q., Zheng Q., Liu X., Weng H., Wu S., Chen W. (2021). Preventing Corneal Blindness Caused by Keratitis Using Artificial Intelligence. Nat. Commun..

[85] Rampat R., Deshmukh R., Chen X., Ting D.S.W., Said D.G., Dua H.S., Ting D.S.J. (2021). Artificial Intelligence in Cornea, Refractive Surgery, and Cataract: Basic Principles, Clinical Applications, and Future Directions. Asia Pac. J. Ophthalmol..

[86] Ting D.S.J., Gopal B.P., Deshmukh R., Seitzman G.D., Said D.G., Dua H.S. (2022). Diagnostic Armamentarium of Infectious Keratitis: A Comprehensive Review. Ocul. Surf..

[87] Konstantopoulos A., Kuo J., Anderson D., Hossain P. (2008). Assessment of the Use of Anterior Segment Optical Coherence Tomography in Microbial Keratitis. Am. J. Ophthalmol..

[88] Konstantopoulos A., Yadegarfar G., Fievez M., Anderson D.F., Hossain P. (2011). In Vivo Quantification of Bacterial Keratitis with Optical Coherence Tomography. Investig. Ophthalmol. Vis. Sci..

[89] Sharma N., Singhal D., Maharana P.K., Agarwal T., Sinha R., Satpathy G., Bageshwar L.M.S., Titiyal J.S. (2018). Spectral Domain Anterior Segment Optical Coherence Tomography in Fungal Keratitis. Cornea.

[90] Ting D.S.J., Ghosh S. (2019). Central Toxic Keratopathy after Contact Lens Wear and Mechanical Debridement: Clinical Characteristics, and Visual and Corneal Tomographic Outcomes. Eye Contact Lens.

[91] Ting D.S.J., Danjoux J.-P. (2019). Late-Onset Traumatic Dislocation of Laser in Situ Keratomileusis Corneal Flaps: A Case Series with Many Clinical Lessons. Int. Ophthalmol..

[92] Takezawa Y., Suzuki T., Shiraishi A. (2017). Observation of Retrocorneal Plaques in Patients with Infectious Keratitis Using Anterior Segment Optical Coherence Tomography. Cornea.

[93] Ting D.S.J., Galal M., Kulkarni B., Elalfy M.S., Lake D., Hamada S., Said D.G., Dua H.S. (2021). Clinical Characteristics and Outcomes of Fungal Keratitis in the United Kingdom 2011–2020: A 10-Year Study. J. Fungi.

[94] Soliman W., Nassr M.A., Abdelazeem K., Al-Hussaini A.K. (2019). Appearance of Herpes Simplex Keratitis on Anterior Segment Optical Coherence Tomography. Int. Ophthalmol..

[95] Soliman W., Fathalla A.M., El-Sebaity D.M., Al-Hussaini A.K. (2013). Spectral Domain Anterior Segment Optical Coherence Tomography in Microbial Keratitis. Graefe’s Arch. Clin. Exp. Ophthalmol..

[96] Yamazaki N., Kobayashi A., Yokogawa H., Ishibashi Y., Oikawa Y., Tokoro M., Sugiyama K. (2014). In Vivo Imaging of Radial Keratoneuritis in Patients with Acanthamoeba Keratitis by Anterior-Segment Optical Coherence Tomography. Ophthalmology.

[97] Park Y.M., Lee J.S., Yoo J.-M., Park J.M., Seo S.-W., Chung I.-Y., Kim S.J. (2018). Comparison of Anterior Segment Optical Coherence Tomography Findings in Acanthamoeba Keratitis and Herpetic Epithelial Keratitis. Int. J. Ophthalmol..

[98] Sridhar M.S., Shaik B. (2018). Anterior Segment Optical Coherence Tomography of Microsporidial Keratoconjunctivitis. Indian. J. Ophthalmol..

[99] Thanathanee O., Laohapitakvorn S., Anutarapongpan O., Suwan-Apichon O., Bhoomibunchoo C. (2019). Anterior Segment Optical Coherence Tomography Images in Microsporidial Keratoconjunctivitis. Cornea.

[100] Yokogawa H., Kobayashi A., Yamazaki N., Sugiyama K. (2014). In Vivo Imaging of Coin-Shaped Lesions in Cytomegalovirus Corneal Endotheliitis by Anterior Segment Optical Coherence Tomography. Cornea.

[101] Kobayashi R., Hashida N., Soma T., Koh S., Miki A., Usui S., Maeda N., Nishida K. (2016). Clinical Findings of Anterior Segment Spectral Domain Optical Coherence Tomography Images in Cytomegalovirus Corneal Endotheliitis. Cornea.

[102] Pinto R.D.P., Lira R.P.C., Arieta C.E.L., de Castro R.S., Bonon S.H.A. (2015). The Prevalence of Adenoviral Conjunctivitis at the Clinical Hospital of the State University of Campinas, Brazil. Clinics.

[103] Sambursky R.P., Fram N., Cohen E.J. (2007). The Prevalence of Adenoviral Conjunctivitis at the Wills Eye Hospital Emergency Room. Optom.-J. Am. Optom. Assoc..

[104] Hoffman J. (2020). Adenovirus: Ocular Manifestations. Community Eye Health.

[105] Pennington M.R., Saha A., Painter D.F., Gavazzi C., Ismail A.M., Zhou X., Chodosh J., Rajaiya J. (2019). Disparate Entry of Adenoviruses Dictates Differential Innate Immune Responses on the Ocular Surface. Microorganisms.

[106] Arici C., Mergen B. (2021). Late-Term Topical Tacrolimus for Subepithelial Infiltrates Resistant to Topical Steroids and Ciclosporin Secondary to Adenoviral Keratoconjunctivitis. Br. J. Ophthalmol..

[107] Gouider D., Khallouli A., Maalej A., Khochtali S., Khairallah M. (2022). Role of Anterior Segment Optical Coherence Tomography in Monitoring Epidemic Keratoconjunctivitis. J. Curr. Ophthalmol..

[108] Royal College of General Practitioners (2001). Royal College of Ophthalmologists (2001) Ophthalmology for General Practice Trainees.

[109] Wong T.Y., Lincoln A., Tielsch J.M., Baker S.P. (1998). The Epidemiology of Ocular Injury in a Major US Automobile Corporation. Eye.

[110] Celebi A.R.C., Kilavuzoglu A.E., Altiparmak U.E., Cosar C.B., Ozkiris A. (2016). The Role of Anterior Segment Optical Coherence Tomography in the Management of an Intra-Corneal Foreign Body. Springerplus.

[111] Armarnik S., Mimouni M., Goldenberg D., Segev F., Meshi A., Segal O., Geffen N. (2019). Characterization of Deeply Embedded Corneal Foreign Bodies with Anterior Segment Optical Coherence Tomography. Graefes Arch. Clin. Exp. Ophthalmol..

[112] Akbaş E., Barut Selver Ö., Palamar M. (2021). Retrospective Evaluation of Corneal Foreign Bodies with Anterior Segment Optical Coherence Tomography. Turk. J. Ophthalmol..

[113] Simon G., Ren Q., Kervick G.N., Parel J.-M. (1993). Optics of the Corneal Epithelium. J. Refract. Surg..

[114] Reinstein D.Z., Silverman R.H., Coleman D.J. (1993). High-Frequency Ultrasound Measurement of the Thickness of the Corneal Epithelium. J. Refract. Surg..

[115] Reinstein D.Z., Archer T.J., Vida R.S. (2022). Applications of Epithelial Thickness Mapping in Corneal Refractive Surgery. Saudi J. Ophthalmol..

[116] Urs R., Lloyd H.O., Reinstein D.Z., Silverman R.H. (2016). Comparison of Very-High-Frequency Ultrasound and Spectral-Domain Optical Coherence Tomography Corneal and Epithelial Thickness Maps. J. Cataract. Refract. Surg..

[117] Li H.F., Petroll W.M., Møller-Pedersen T., Maurer J.K., Cavanagh H.D., Jester J.V. (1997). Epithelial and Corneal Thickness Measurements by In Vivo Confocal Microscopy through Focusing (CMTF). Curr. Eye Res..

[118] Sella R., Zangwill L.M., Weinreb R.N., Afshari N.A. (2019). Repeatability and Reproducibility of Corneal Epithelial Thickness Mapping with Spectral-Domain Optical Coherence Tomography in Normal and Diseased Cornea Eyes. Am. J. Ophthalmol..

[119] Ma J.X., Wang L., Weikert M.P., Montes de Oca I., Koch D.D. (2019). Evaluation of the Repeatability and Reproducibility of Corneal Epithelial Thickness Mapping for a 9-Mm Zone Using Optical Coherence Tomography. Cornea.

[120] Khamar P., Rao K., Wadia K., Dalal R., Grover T., Versaci F., Gupta K. (2020). Advanced Epithelial Mapping for Refractive Surgery. Indian J. Ophthalmol..

[121] Haque S., Simpson T., Jones L. (2006). Corneal and Epithelial Thickness in Keratoconus: A Comparison of Ultrasonic Pachymetry, Orbscan II, and Optical Coherence Tomography. J. Refract. Surg..

[122] Reinstein D.Z., Gobbe M., Archer T.J., Couch D. (2011). Epithelial Thickness Profile as a Method to Evaluate the Effectiveness of Collagen Cross-Linking Treatment After Corneal Ectasia. J. Refract. Surg..

[123] Kanellopoulos A.J., Asimellis G. (2014). In Vivo 3-Dimensional Corneal Epithelial Thickness Mapping as an Indicator of Dry Eye: Preliminary Clinical Assessment. Am. J. Ophthalmol..

[124] Ondas O., Keles S. (2014). Central Corneal Thickness in Patients with Atopic Keratoconjunctivitis. Med. Sci. Monit. Int. Med. J. Exp. Clin. Res..

[125] Buffault J., Zéboulon P., Liang H., Chiche A., Luzu J., Robin M., Rabut G., Labetoulle M., Labbé A., Baudouin C. (2020). Assessment of Corneal Epithelial Thickness Mapping in Epithelial Basement Membrane Dystrophy. PLoS ONE.

[126] Levy A., Georgeon C., Knoeri J., Tourabaly M., Leveziel L., Bouheraoua N., Borderie V.M. (2022). Corneal Epithelial Thickness Mapping in the Diagnosis of Ocular Surface Disorders Involving the Corneal Epithelium: A Comparative Study. Cornea.

[127] Seitz B., Asi F., Mäurer S., Hamon L., Quintin A., Langenbucher A. (2021). Anterior Segment OCT: Application to Improve Graft Selection for Corneal Transplantation. Atlas of Anterior Segment Optical Coherence Tomography.

[128] Ting D.S.J., Sau C.Y., Srinivasan S., Ramaesh K., Mantry S., Roberts F. (2012). Changing Trends in Keratoplasty in the West of Scotland: A 10-Year Review. Br. J. Ophthalmol..

[129] Dunker S.L., Veldman M.H.J., Winkens B., van den Biggelaar F.J.H.M., Nuijts R.M.M.A., Kruit P.J., Dickman M.M., Dutch Cornea Consortium (2021). Real-World Outcomes of DMEK: A Prospective Dutch Registry Study. Am. J. Ophthalmol..

[130] Zhang A.Q., Rubenstein D., Price A.J., Côté E., Levitt M., Sharpen L., Slomovic A. (2013). Evolving Surgical Techniques of and Indications for Corneal Transplantation in Ontario: 2000–2012. Can. J. Ophthalmol..

[131] Ting D.S.J., Deshmukh R., Ting D.S.W., Ang M. (2023). Big Data in Corneal Diseases and Cataract: Current Applications and Future Directions. Front. Big Data.

[132] Liu S., Wong Y.L., Walkden A. (2022). Current Perspectives on Corneal Transplantation. Clin. Ophthalmol..

[133] Kaiserman I., Bahar I., Rootman D.S. (2008). Corneal Wound Malapposition after Penetrating Keratoplasty: An Optical Coherence Tomography Study. Br. J. Ophthalmol..

[134] Weller J.M., Hübner L., Kruse F.E., Tourtas T. (2023). Characterisation of Ectasia after Penetrating Keratoplasty in Keratoconus Eyes Using Anterior Segment Optical Coherence Tomography. Br. J. Ophthalmol..

[135] Niederkorn J.Y., Larkin D.F.P. (2010). Immune Privilege of Corneal Allografts. Ocul. Immunol. Inflamm..

[136] Ong Z.Z., Wong T.L., Suresh L., Hammoudeh Y., Lister M., Said D.G., Dua H.S., Ting D.S.J. (2023). A 7-Year Review of Clinical Characteristics, Predisposing Factors and Outcomes of Post-Keratoplasty Infectious Keratitis: The Nottingham Infectious Keratitis Study. Front. Cell Infect. Microbiol..

[137] Gómez-Benlloch A., Montesel A., Pareja-Aricò L., Mingo-Botín D., Michael R., Barraquer R.I., Alió J. (2021). Causes of Corneal Transplant Failure: A Multicentric Study. Acta Ophthalmol..

[138] Larkin D.F. (1994). Corneal Allograft Rejection. Br. J. Ophthalmol..

[139] Yenerel N.M., Kucumen R.B., Gorgun E. (2013). The Complementary Benefit of Anterior Segment Optical Coherence Tomography in Penetrating Keratoplasty. Clin. Ophthalmol..

[140] Anwar M., Teichmann K.D. (2002). Big-Bubble Technique to Bare Descemet’s Membrane in Anterior Lamellar Keratoplasty. J. Cataract. Refract. Surg..

[141] Wylegała E., Nowińska A. (2009). Usefulness of Anterior Segment Optical Coherence Tomography in Descemet Membrane Detachment. Eur. J. Ophthalmol..

[142] Chamberlain W., Austin A., Terry M., Jeng B.H., Rose-Nussbaumer J. (2016). Survey of Experts on Current Endothelial Keratoplasty Techniques. J. Clin. Exp. Ophthalmol..

[143] Price M.O., Giebel A.W., Fairchild K.M., Price F.W. (2009). Descemet’s Membrane Endothelial Keratoplasty: Prospective Multicenter Study of Visual and Refractive Outcomes and Endothelial Survival. Ophthalmology.

[144] Ham L., Dapena I., van Luijk C., van der Wees J., Melles G.R.J. (2009). Descemet Membrane Endothelial Keratoplasty (DMEK) for Fuchs Endothelial Dystrophy: Review of the First 50 Consecutive Cases. Eye.

[145] Vasiliauskaite I., Kocaba V., van Dijk K., Baydoun L., Lanser C., Lee D., Jager M.J., Melles G.R.J., Oellerich S. (2023). Long-Term Outcomes of Descemet Membrane Endothelial Keratoplasty: Effect of Surgical Indication and Disease Severity. Cornea.

[146] Ang M., Ting D.S.J., Kumar A., May K.O., Htoon H.M., Mehta J.S. (2020). Descemet Membrane Endothelial Keratoplasty in Asian Eyes: Intraoperative and Postoperative Complications. Cornea.

[147] Lee W.B., Jacobs D.S., Musch D.C., Kaufman S.C., Reinhart W.J., Shtein R.M. (2009). Descemet’s Stripping Endothelial Keratoplasty: Safety and Outcomes: A Report by the American Academy of Ophthalmology. Ophthalmology.

[148] Dirisamer M., van Dijk K., Dapena I., Ham L., Oganes O., Frank L.E., Melles G.R.J. (2012). Prevention and Management of Graft Detachment in Descemet Membrane Endothelial Keratoplasty. Arch. Ophthalmol..

[149] Deshmukh R., Nair S., Ting D.S.J., Agarwal T., Beltz J., Vajpayee R.B. (2022). Graft Detachments in Endothelial Keratoplasty. Br. J. Ophthalmol..

[150] Tey K.Y., Tan S.Y., Ting D.S.J., Mehta J.S., Ang M. (2022). Effects of Combined Cataract Surgery on Outcomes of Descemet’s Membrane Endothelial Keratoplasty: A Systematic Review and Meta-Analysis. Front. Med..

[151] Coco G., Levis H.J., Borgia A., Romano D., Pagano L., Virgili G., Kaye S.B., Romano V. (2023). Posterior Stromal Ripples Increase Risk of Descemet’s Membrane Endothelial Keratoplasty Graft Detachment Worsening over Time. Acta Ophthalmol..

[152] Fernández López E., Baydoun L., Gerber-Hollbach N., Dapena I., Liarakos V.S., Ham L., Melles G.R.J. (2016). Rebubbling Techniques for Graft Detachment after Descemet Membrane Endothelial Keratoplasty. Cornea.

[153] Yeh R.-Y., Quilendrino R., Musa F.U., Liarakos V.S., Dapena I., Melles G.R.J. (2013). Predictive Value of Optical Coherence Tomography in Graft Attachment after Descemet’s Membrane Endothelial Keratoplasty. Ophthalmology.

[154] Ting D.S.J., Said D.G., Dua H.S. (2019). Interface Haze after Descemet Stripping Automated Endothelial Keratoplasty. JAMA Ophthalmol..

[155] Song A., Deshmukh R., Lin H., Ang M., Mehta J.S., Chodosh J., Said D.G., Dua H.S., Ting D.S.J. (2021). Post-Keratoplasty Infectious Keratitis: Epidemiology, Risk Factors, Management, and Outcomes. Front. Med..

[156] Sharma N., Kaur M., Titiyal J.S., Aldave A. (2021). Infectious Keratitis after Lamellar Keratoplasty. Surv. Ophthalmol..

[157] Market Scope: Refractive Surgery to Grow 9.6% a Year through 2025, Despite COVID-19. https://eyewire.news/news/market-scope-refractive-surgery-to-grow-9-6-a-year-through-2025-despite-covid-19.

[158] Sutton G.L., Kim P. (2010). Laser in Situ Keratomileusis in 2010—A Review. Clin Exp Ophthalmol..

[159] Sandoval H.P., Donnenfeld E.D., Kohnen T., Lindstrom R.L., Potvin R., Tremblay D.M., Solomon K.D. (2016). Modern Laser in Situ Keratomileusis Outcomes. J. Cataract. Refract. Surg..

[160] Pallikaris I.G., Papatzanaki M.E., Stathi E.Z., Frenschock O., Georgiadis A. (1990). Laser in Situ Keratomileusis. Lasers Surg. Med..

[161] Buratto L., Ferrari M. (1992). Excimer Laser Intrastromal Keratomileusis: Case Reports. J. Cataract. Refract. Surg..

[162] Dawson D.G., Schmack I., Holley G.P., Waring G.O., Grossniklaus H.E., Edelhauser H.F. (2007). Interface Fluid Syndrome in Human Eye Bank Corneas after LASIK: Causes and Pathogenesis. Ophthalmology.

[163] Lyle W.A., Jin G.J. (1999). Interface Fluid Associated with Diffuse Lamellar Keratitis and Epithelial Ingrowth after Laser in Situ Keratomileusis. J. Cataract. Refract. Surg..

[164] Galvis V., Berrospi R.D., Tello A., Santaella G. (2019). Interface Fluid Syndrome (IFS) Following Toxic Anterior Segment Syndrome (TASS): Not Related to High Intraocular Pressure but to Endothelial Failure. Saudi J. Ophthalmol..

[165] Rosas Salaroli C.H., Li Y., Huang D. (2009). High-Resolution Optical Coherence Tomography Visualization of LASIK Flap Displacement. J. Cataract. Refract. Surg..

[166] Ting D.S.J., Srinivasan S., Danjoux J.-P. (2018). Epithelial Ingrowth Following Laser in Situ Keratomileusis (LASIK): Prevalence, Risk Factors, Management and Visual Outcomes. BMJ Open Ophthalmol..

[167] Kim H., Lim M.C., Mannis M.J., Kim E.S. (2019). Epithelial Downgrowth after Femtosecond Laser-Assisted Cataract Surgery. Am. J. Ophthalmol. Case Rep..

[168] Maumenee A.E., Paton D., Morse P.H., Butner R. (1970). Review of 40 Histologically Proven Cases of Epithelial Downgrowth Following Cataract Extraction and Suggested Surgical Management. Am. J. Ophthalmol..

[169] Sugar A., Meyer R.F., Hood C.I. (1977). Epithelial Downgrowth Following Penetrating Keratoplasty in the Aphake. Arch. Ophthalmol..

[170] Weiner M.J., Trentacoste J., Pon D.M., Albert D.M. (1989). Epithelial Downgrowth: A 30-Year Clinicopathological Review. Br. J. Ophthalmol..

[171] Randleman J.B., Russell B., Ward M.A., Thompson K.P., Stulting R.D. (2003). Risk Factors and Prognosis for Corneal Ectasia after LASIK. Ophthalmology.

[172] Binder P.S., Lindstrom R.L., Stulting R.D., Donnenfeld E., Wu H., McDonnell P., Rabinowitz Y. (2005). Keratoconus and Corneal Ectasia after LASIK. J. Cataract. Refract. Surg..

[173] Goebels S., Eppig T., Wagenpfeil S., Cayless A., Seitz B., Langenbucher A. (2017). Complementary Keratoconus Indices Based on Topographical Interpretation of Biomechanical Waveform Parameters: A Supplement to Established Keratoconus Indices. Comput. Math. Methods Med..

[174] Ambrósio R., Dawson D.G., Salomão M., Guerra F.P., Caiado A.L.C., Belin M.W. (2010). Corneal Ectasia after LASIK despite Low Preoperative Risk: Tomographic and Biomechanical Findings in the Unoperated, Stable, Fellow Eye. J. Refract. Surg..

[175] Klein S.R., Epstein R.J., Randleman J.B., Stulting R.D. (2006). Corneal Ectasia after Laser in Situ Keratomileusis in Patients without Apparent Preoperative Risk Factors. Cornea.

[176] Reinstein D.Z., Gobbe M., Archer T.J., Youssefi G., Sutton H.F.S. (2015). Stromal Surface Topography-Guided Custom Ablation as a Repair Tool for Corneal Irregular Astigmatism. J. Refract. Surg..

[177] Huang D., Tang M., Shekhar R. (2003). Mathematical Model of Corneal Surface Smoothing after Laser Refractive Surgery. Am. J. Ophthalmol..

[178] Silverman R.H., Urs R., RoyChoudhury A., Archer T.J., Gobbe M., Reinstein D.Z. (2014). Epithelial Remodeling as Basis for Machine-Based Identification of Keratoconus. Investig. Ophthalmol. Vis. Sci..

[179] Reinstein D.Z., Archer T.J., Gobbe M., Silverman R.H., Coleman D.J. (2008). Epithelial Thickness in the Normal Cornea: Three-Dimensional Display With Very High Frequency Ultrasound. J. Refract. Surg..

[180] Gaster R.N., Canedo A.L.C., Rabinowitz Y.S. (2013). Corneal Collagen Cross-Linking for Keratoconus and Post-LASIK Ectasia. Int. Ophthalmol. Clin..

[181] Li Y., Tan O., Brass R., Weiss J.L., Huang D. (2012). Corneal Epithelial Thickness Mapping by Fourier-Domain Optical Coherence Tomography in Normal and Keratoconic Eyes. Ophthalmology.

[182] Hwang E.S., Schallhorn J.M., Randleman J.B. (2020). Utility of Regional Epithelial Thickness Measurements in Corneal Evaluations. Surv. Ophthalmol..

[183] Maslin J.S., Barkana Y., Dorairaj S.K. (2015). Anterior Segment Imaging in Glaucoma: An Updated Review. Indian. J. Ophthalmol..

[184] Ting D.S.J., Foo V.H., Yang L.W.Y., Sia J.T., Ang M., Lin H., Chodosh J., Mehta J.S., Ting D.S.W. (2021). Artificial Intelligence for Anterior Segment Diseases: Emerging Applications in Ophthalmology. Br. J. Ophthalmol..

